# Multi-omics reveal immune microenvironment alterations in multiple myeloma and its precursor stages

**DOI:** 10.1038/s41408-024-01172-x

**Published:** 2024-11-06

**Authors:** Yan Cheng, Fumou Sun, Daisy V. Alapat, Visanu Wanchai, David Mery, Eric R. Siegel, Hongwei Xu, Sarah Johnson, Wancheng Guo, Clyde Bailey, Cody Ashby, Michael Anton Bauer, Samer Al Hadidi, Carolina Schinke, Sharmilan Thanendrarajan, Maurizio Zangari, Frits van Rhee, Guido Tricot, John D. Shaughnessy, Fenghuang Zhan

**Affiliations:** 1 Myeloma Center, Winthrop P. Rockefeller Institute, Department of Internal Medicine, University of Arkansas for Medical Sciences, Little Rock, AR 72205 USA; 2 Department of Pathology, College of Medicine, University of Arkansas for Medical Sciences, Little Rock, AR 72205 USA; 3 Department of Biostatistics, University of Arkansas for Medical Sciences, Little Rock, AR 72205 USA; 4 Department of Biomedical Informatics, College of Medicine, University of Arkansas for Medical Sciences, Little Rock, AR 72205 USA

**Keywords:** Cancer microenvironment, Myeloma

## Abstract

Tumor immune microenvironmental alterations occur early in multiple myeloma (MM) development. In this study, we aim to systematically characterize the tumor immune microenvironment (TME) and the tumor-immune interactions from precursor stages, i.e., monoclonal gammopathy of undetermined significance (MGUS) and smoldering MM (SMM), to newly diagnosed MM, comparing these to healthy donors. Using CIBERSORT, mass cytometry (CyTOF), and single-cell RNA sequencing (scRNA-Seq), we examined innate and adaptive immune changes across these stages. We found a decrease in granulocytes in the TME predicts MM outcomes. HLA-DR is reduced in CD16^+^ monocytes and plasmacytoid dendritic cells, while myeloid dendritic cells show decreased expression of stress and immune-response genes. NK cells and CD8^+^ T cells shift from a GZMK^+^ to a GZMB^+^ cytotoxic phenotype in the TME, with increased inhibitory markers TIM3 and TIGIT. In paired samples, the proportion and gene expression pattern in patient-specific GZMB^+^CD8^+^ T cells remain largely unchanged despite MM progression. Our findings provide a comprehensive immune landscape of MM and its precursors, offering insights into therapeutic strategies. Enhancing neutrophil and NK cell cytotoxicity, tumor antigen presentation, and CD8^+^ T cell versatility in precursor stages may prevent MM progression.

## Introduction

Multiple myeloma (MM) is characterized by uncontrolled clonal proliferation of plasma cells in the bone marrow (BM) [1]. The disease progresses from the precursor stages, monoclonal gammopathy of undetermined significance (MGUS) and smoldering MM (SMM) with average progression rates at 1% and 10% per year during the first 5 years, respectively [2, 3]. Since the precursor stages of MM are asymptomatic and not all cases progress, treatment is not routinely administered [4, 5]. Gene expression profiles (GEP) of purified tumor cells have revealed that all molecular subtypes of MM are also present in the precursor stages [6]. Other genomic studies have identified genomic aberrations that differentiate progressive and stable MM precursor conditions, e.g., del17p13 (*TP53*), IgH translocations, gain 1q, mutations in the MAPK pathway and translocations involving *MYC* [7, 8]. However, not all patients with these genomic aberrations eventually progress to MM [9], suggesting that tumor-extrinsic factors, such as the tumor immune microenvironment, may also play a significant role.

Understanding of the immune system in MM [10] has been accompanied by transformative advances in the therapeutic landscape, leading to major improvements in patient outcomes. FDA-approved B-cell maturation antigen (BCMA)-targeting CAR T cell immunotherapies [11], monoclonal antibodies that target CD38 [12, 13] or BCMA [14, 15], and bispecific T cell engager (BiTE) antibody [16–18] have achieved great success in relapsed and refractory MM. Recent investigations using single-cell-based technologies have shown substantial alterations in the tumor microenvironment (TME) in MM and its precursor stages [19–21], such as increased percentages of NK cells, T cells, and non-classical monocytes. Whether immune microenvironmental alterations drive MM progression from its precursor stages or merely serve as surrogate markers of increased tumor burden is still unclear. The PVX-410 vaccine, mimicking three MM-associated antigens, X-box binding protein 1 (XBP1), syndecan-1 (CD138), and SLAM family member 7 (SLAMF7), has shown safety and immunogenicity in SMM patients, and more novel vaccines are under investigation in SMM and MGUS patients [22–25]. Deep molecular characterization of the immune profiles and their crosstalk with tumor cells in MM and its precursor conditions is needed to advance treatment development and discover safe and efficient strategies to prevent MGUS and SMM progression.

In this study, we aim to systematically characterize the TME and the tumor-immune interactions in MGUS, SMM, and NDMM. We evaluated the BM cell content using a whole BM gene expression-based computational technique, CIBERSORT [26–28], and investigated the correlation of immune cell proportions with patient outcome after 5-20 years of follow-up. We also analyzed BM immune cell functional marker expression using CyTOF in a large cohort of patients and examined BM immune cell gene expression in paired stable or progressive MM precursor conditions and MM samples using single-cell RNA sequencing (scRNA-Seq). By integrating results from CIBERSORT, CyTOF, and scRNA-Seq, our study provides a comprehensive map of innate and adaptive immune cell alterations during progression from precursor conditions to MM. This information should help develop strategies for immune-based prevention of MM progression.

## Results

### A decreased granulocyte proportion predicts a poor outcome in NDMM

**Fig. 1:**
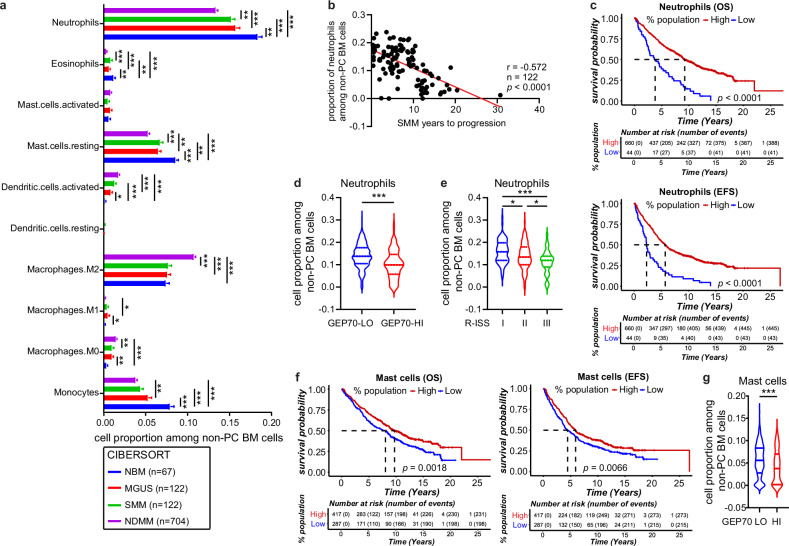
A decreased neutrophil proportion predicts a poor outcome. **a** Bar plot showing proportions of the 10 innate immune cell types of NBM (normal bone marrow, *n* = 67), MGUS (monoclonal gammopathy of unknown significance, *n* = 122), SMM (smoldering multiple myeloma, *n* = 122), and NDMM (newly diagnosed multiple, *n* = 704) from CIBERSORT analysis. Error bars represent mean ± standard error mean (SEM). *p* values for each cell type were calculated using Kruskal–Wallis with Dunn’s multiple comparisons test. **b** Correlation analysis of neutrophil proportions with the time of progression in SMM patients. Pearson *r* = -0.572, *p* < 0.0001. **c** Kaplan–Meier analyses of overall survival (OS, up) and event-free survival (EFS, down) in MM patients with high and low neutrophil proportions. Hazard ratios OS = 22.69, *p* < 0.0001; Hazard ratios EFS = 2.59, *p* < 0.0001. High vs. Low groups were determined by the optimal cut point of EFS survival. **d** Violin plot showing the proportions of neutrophils in MM patients with GEP70-Low (*n* = 598) and GEP70-High (*n* = 105) (GEP70 cut off 0.66). *p* values were calculated using Mann–Whitney test. **e** Violin plot showing the proportions of neutrophils in MM patients in R-ISS stages I (*n* = 88), II (*n* = 193), and III (*n* = 42) (right). *p* values were calculated using the One-way ANOVA with Tukey’s multiple comparisons test. **f** Kaplan–Meier analyses of overall survival (OS, left) and event-free survival (EFS, right) in MM patients with high and low mast cell proportions. Hazard ratios OS = 1.35, *p* = 0.0018; Hazard ratios EFS = 1.28, *p* = 0.0068. High vs. Low groups were determined by the optimal cut point of EFS survival. **g** Violin plot showing the proportions of mast cells in MM patients with GEP70-Low (*n* = 598) and GEP70-High (*n* = 105) (GEP70 cut off 0.66). *p* values were calculated using Mann–Whitney test.

To better understand the characteristics of the BM microenvironment during MM progression, we applied MGSM27 signature matrix, a gene-expression–based computational technique [27], to mRNA gene expression profiles (GEP) of BM biopsy from patients with MGUS (*n* = 122), SMM (*n* = 122) and NDMM (*n* = 704), as well as healthy donors (normal BM (NBM), *n* = 67) (Supplementary Tables 1). This so-called CIBERSORT [26, 28] algorithm reconstructed 27 BM cell types and revealed the estimated cell proportions from the BM biopsy. We removed plasma cells (PC) from the 100% total cell proportions to avoid bias resulting from different tumor burdens. CIBERSORT revealed six types of innate immune cells, neutrophils, eosinophils, mast cells, dendritic cells (DCs), macrophages, and monocytes. It revealed that the proportions of neutrophils, mast cells, and monocytes account for the majority of innate immune cells and were decreased in NDMM and its precursor stages when compared with NBM (Fig. 1a). We noticed a 45% decrease of M2 macrophages in NDMM compared with NDMM, but not in the precursor stages (Fig. 1a). We performed correlation analysis of immune cell proportions with the time of progression of MGUS and SMM patients. We observed high neutrophil proportions correlated with short progression time in SMM patients (Fig. 1b). However, NDMM patients with high proportions of neutrophils had superior overall survival (OS) and event-free survival (EFS) (Fig. 1c). In support of that, the neutrophil percentage was decreased in patients with high-risk status based on the 70-gene Prognostic Risk Score (GEP-70) or at stage III of International Staging System (ISS) (Fig. 1d, e). We observed similar findings related to mast cells (Fig. 1f, g), indicating that a decreasing proportion of granulocytes predicts inferior survival in NDMM patients. In addition, MM patients with high-risk GEP-70 had a higher M2 macrophage proportion (Supplementary Fig. 1a). M2 macrophages were increased in MM patients with ISS stage III (Supplementary Fig. 1b). Collectively, these data suggest that the alterations of innate immune cells proportion start early in MGUS stage and are predictive of outcome. Notably, we observed MM patients with high or low MM plasma cell percentages displaying distinct immune signatures and that the immune signatures in MM patients with low MM plasma cell percentages resembled that of MGUS or SMM.

### Reduced HLA-DR surface expression in CD16^+^ monocytes and pDCs is detected early at the MGUS stage

**Fig. 2:**
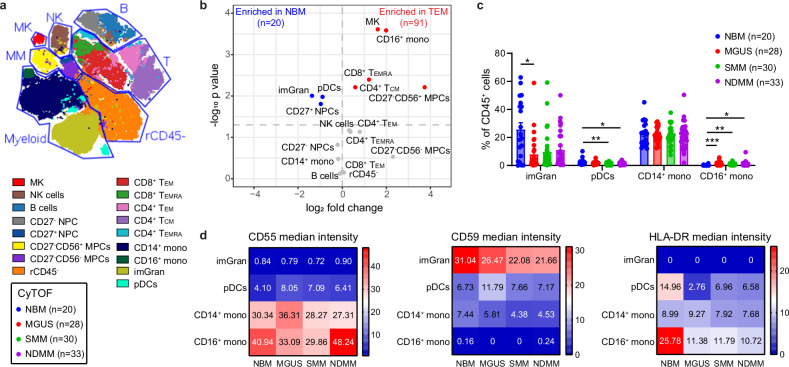
Reduced HLA-DR surface expression in CD16^+^ monocytes and pDCs is detected early at the MGUS stage. **a** viSNE plot showing 17 metaclusters (MC) in the normal bone marrow (NBM) and tumor microenvironment (TME) were identified by FlowSOM analysis. **b** Volcano plot showing MC changes between NBM and TME. For each cell type the log fold change in mean cell fraction between tumor and normal samples, with −log_10_ Benjamin–Hochberg-corrected, two-sided Wilcoxon rank-sum *p* values on the *y*-axis is shown. **c** Bar plot showing innate cell composition changes among different disease stages. *p* values for each cell type were calculated using Kruskal–Wallis with Dunn’s multiple comparisons test. **d** Heatmap showing median intensity of indicated channels in each innate immune cell types. *p* values for each cell type were calculated using Kruskal–Wallis with Dunn’s multiple comparisons test.

To evaluate the immune cell ratio and function based on protein expression, we performed deep phenotyping of BM aspirates of patients with MGUS (*n* = 28), SMM (*n* = 30), NDMM (*n* = 33), as well as 20 NBM using CyTOF (Supplementary Tables 2, 3). Clustering based on cell lineage marker expression using the FlowSOM algorithm [29] identified 17 metaclusters (MCs, Fig. 2a, Supplementary Fig. 2a). We observed an enrichment of megakaryocytes, CD16^+^ monocytes, CD8^+^ Temra (terminally differentiated effector), CD4^+^ Tcm (central memory), and CD56^+^ MM in the TME, as well as a decrease of plasmacytoid DCs (pDCs), immature granulocytes, and CD27^+^ normal plasma cells (NPC) (Fig. 2b). In addition to these immune cell alterations, we also observed an enrichment of NK cells, CD4^+^ Tem (effector memory) and CD4^+^ Temra in the TME after excluding the two malignant plasma cell (MPC) clusters (Supplementary Fig. 3). CyTOF showed 65% decrease of an immature granulocytes in the MGUS stage compared to NBM, 53% decrease of pDCs in SMM and 51% decrease in NDMM compared to NBM, and 5.6-fold increase of CD16^+^ monocytes in MGUS, 2.0 -fold increase in SMM and 1.6-fold increase in NDMM compared with NBM (Fig. 2c). Next, we investigated the functional marker expression among innate immune cells. Complement inhibitory proteins CD55 and CD59 showed cell type specificity in monocytes and immature granulocytes, respectively, but only CD55 showed increased intensity in MM compared with its precursor stages (Fig. 2d). A decrease of HLA-DR expression was observed in pDCs and CD16^+^ monocytes in both MM and its precursor stages (Fig. 2d), indicating that a decrease of myeloid cell antigen presentation function begins early in the MGUS stage.

### Decreased numbers of mDCs and their expression of stress and immune response genes are observed in MM and its precursor stages

**Fig. 3:**
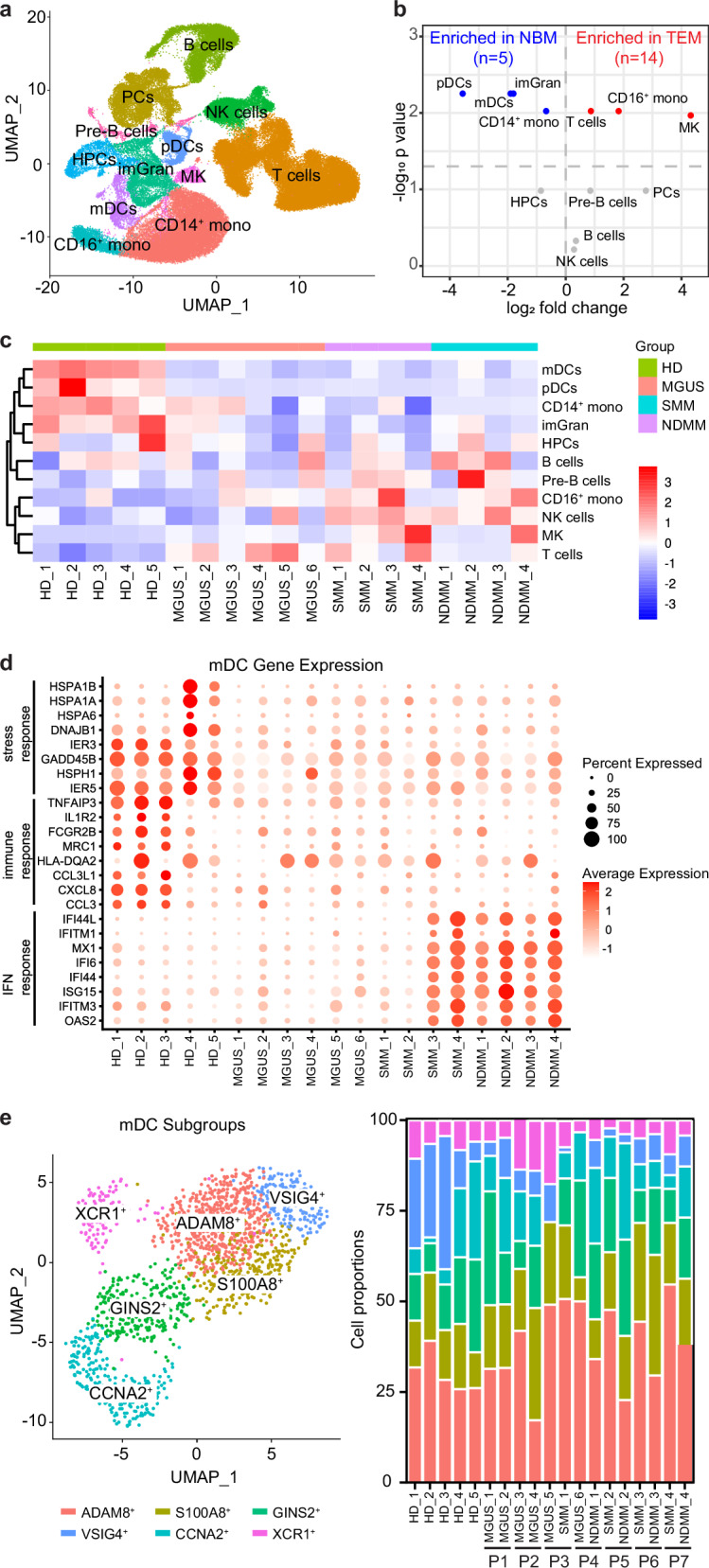
Decreased numbers of mDCs and their expression of stress and immune response genes is observed in MM and its precursor stages. **a** UMAP plot showing 12 cell clusters identified in the BM aspirate samples. **b** Volcano plot showing cell cluster changes between normal bone marrow (NBM) and tumor microenvironment (TME). For each cell type the log fold change in mean cell fraction between tumor and normal samples, with −log_10_ Benjamin-Hochberg-corrected, two-sided Wilcoxon rank-sum *p* values on the *y*-axis is shown. **c** Heatmap showing the fractions of different cell clusters in non-tumor BM cells for individual patients and healthy donors. Cell cluster fractions are z-standardized across patients. **d** Dot plot showing gene expression in myeloid dendritic cells for individual patients and healthy donors. **e** UMAP plots of mDC subtypes (left) and stacked box plots showing their proportions (right). *p* values for each cell subtype were calculated using Benjamin–Hochberg corrected, two-sided Wilcoxon rank sum test.

To reduce the interference of individual heterogeneity and to further explore gene expression associated with immune cell functional alterations during disease progression, we collected sequential BM aspirate samples that consisted of two pairs of stable MGUS samples, one MGUS progressed to SMM, one MGUS progressed to MM, and 3 pairs of SMM progressed to MM, which include a total of 6 MGUS, 4 SMM, and 4 MM, along with 5 NBM controls (Supplementary Tables 4), and performed single-cell RNA sequencing (scRNA-Seq; 10x Genomics). By clustering cells based on gene expression, we identified 12 cell clusters (Fig. 3a, Supplementary Tables 5). Consistent with previous data [20], we observed a decrease in pDCs, myeloid DCs (mDCs), immature granulocytes, and CD14^+^ monocytes in MM and its precursor stages, as well as an increase in T cells, CD16^+^ monocytes, megakaryocytes both before and after excluding PCs (Fig. 3b, Supplementary Fig. 4). MM and NBM showed a distinct pattern of immune cell cluster distribution among total immune cells, with an overall decrease of the proportion of innate immune cell clusters and an increase of NK and T cells (Fig. 3c). The stable MGUS pairs resembled NBM in terms of the proportion of CD14^+^ monocytes, immature granulocytes and NK cells when compared to the progressed MGUS and SMM samples (Fig. 3c).

As myeloid DCs showed a significant decrease in cell number and proportion between NBM and tumor microenvironment (TME), we further explored the alterations of their gene expression among patients and healthy donors. We found the upregulated genes were highly enriched in IFN-responsive pathway (*OAS2, IFITM3, ISG15, IFI44*) and showed increased expression in all MM (4/4) and 2/4 SMM samples, whereas all MGUS samples (6/6) remain unchanged. The downregulated genes were highly enriched for protein folding and cellular stress response (*HSPA1B, HSPA1A, HSPA6, DNAJB1*), inflammation (*TNFAIP3, IL1R2, FCGR2B*), antigen presentation (*MRC1, HLA-DQA2*), and immune cell recruitment and activation (*CCL3L1, CXCL8, CCL3*) in TME (Fig. 3d, Supplementary Fig. 5a). These findings suggest that mDCs in the TME have a decreased ability to respond to cellular stress and to execute their immune function. Subclustering of mDCs based on their gene expression revealed 6 mDC subsets, which were annotated by their marker genes (*ADAM8*^*+*^*, S100A8*^*+*^*, GINS2*^*+*^*, CCNA2*^*+*^*, VSIG4*^*+*^*, and XCR1*^*+*^, Fig. 3e, Supplementary Fig. 5b). The proportion of *VSIG4*^*+*^ mDC subset showed a trend of decrease in the TME (Fig. 3e). VSIG4, a B7 family related protein, is a negative regulator of T cell activation [30]. Whether mDCs in MM and its precursor stages exhibit impaired function in regulating T cell activation needs further investigation.

### NK cells are overactivated and exhausted in TME, and are characterized by high levels of CD57, GZMB, and TIM3

**Fig. 4:**
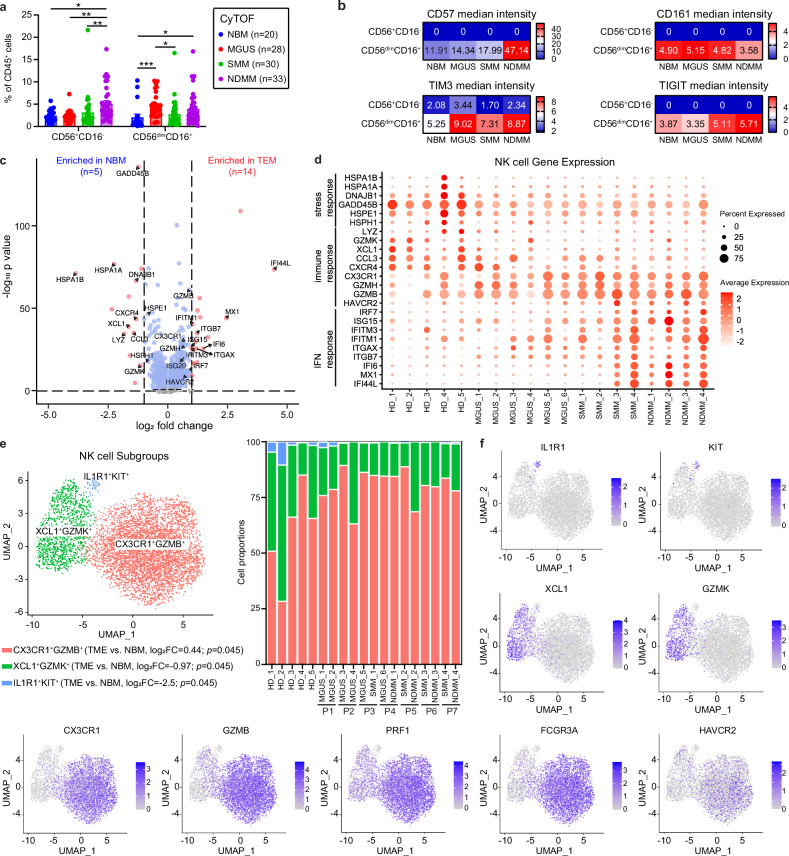
NK cells are overactivated and exhausted in the TME, characterized by high levels of CD57, GZMB and TIM3. **a** Box plot showing NK cell composition changes among different disease stages. *p* values for each cell type were calculated using Kruskal–Wallis with Dunn’s multiple comparisons test. **b** Heatmap showing median intensity of indicated channels in each NK cell types. *p* values for each cell type were calculated using Kruskal–Wallis with Dunn’s multiple comparisons test. **c** Volcano plot showing differential gene expression in immature granulocytes between normal bone marrow (NBM) and tumor microenvironment (TME). **d** Dot plot showing gene expression of NK cells for individual patients and healthy donors. **e** UMAP plots of NK cell subtypes (left) and stacked box plots showing their proportions (right). *p* values for each cell subtype were calculated using Benjamin–Hochberg corrected, two-sided Wilcoxon rank sum test. **f** Density map on the UMAP plot showing gene expression levels among NK subtypes.

Next, we examined NK cell proportion and function during MM progression. CyTOF analysis showed an increase of immunomodulatory (CD56^+^CD16^-^) NK cells in NDMM compared to other stages of disease, while cytolytic (CD56^dim^CD16^+^) NK cells were increased in both MGUS and NDMM compared to NBM (Fig. 4a). Both cytotoxic markers (CD57 and CD161) and immune inhibitory checkpoints (TIM3 and TIGIT) had increased intensity among the cytolytic NK cells. CD57, also a marker of NK cell terminal differentiation, anergy and senescence [31], was increased in NDMM compared with other groups. TIM3 was increased in MGUS, SMM, and MM compared with NBM, and TIGIT was elevated in SMM and NDMM compared with NBM and MGUS (Fig. 4b).

Next, we investigated NK cell clusters using scRNA-Seq analysis. Similar to what we found in the mDCs, gene expression of NK cells showed an upregulation of IFN response genes and a downregulation of stress response genes in the TME compared with NBM (Fig. 4c, d). Interestingly, with regards to the immune response genes, we observed a decrease of *LYZ, GZMK, XCL1, CCL3, CXCR4*, and an increase of *CX3CR1, GZMH, GZMB, HAVCR2* (encoding TIM3) in the TME (Fig. 4c, d), indicating a switch of immune response features of NK cells during MM progression. Subclustering of NK cells revealed three populations, *CX3CR1*^*+*^*GZMB*^*+*^*, XCL1*^*+*^*GZMK*^*+*^, and a less frequent *IL1R1*^*+*^*KIT*^*+*^ subset (Fig. 4e, f). The *IL1R1*^*+*^*KIT*^*+*^ NK cell subset expressed high levels of *TCF7, SELL*, and *IL7R*, indicating those cells were naïve. The *CX3CR1*^*+*^*GZMB*^*+*^ NK cell subset was also characterized by cell surface marker *FCGR3A*^*+*^ (encoding CD16a), high levels of cytolytic genes (*GZMH, PRF1*), and the exhaustion marker *HAVCR2* (encoding TIM3) (Fig. 4e, f). Notably, we observed a proportion shift from the *IL1R1*^*+*^*KIT*^*+*^ and *XCL1*^*+*^*GZMK*^*+*^ NK cell subsets towards the *CX3CR1*^*+*^*GZMB*^*+*^ NK subset in the TME compared with NBM (Fig. 4e, f). Collectively, CyTOF and scRNA-Seq data suggested that despite an increase in the proportion of cytolytic NK cells in the TME, they may be overactivated and thus express exhaustion markers.

### Accumulation of PD1^+^CD4^+^ and TIGIT^+^CD8^+^ inhibitory T cells during MM progression

**Fig. 5:**
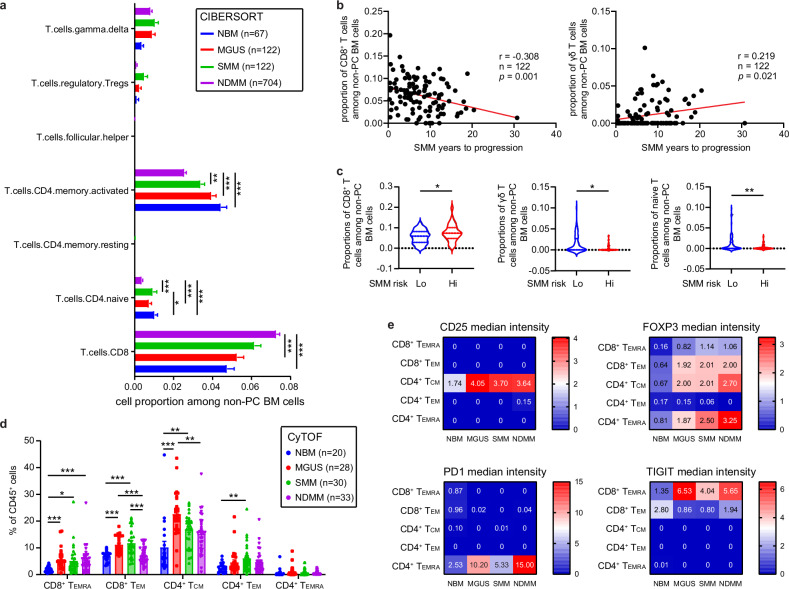
Accumulation of PD1^+^CD4 and TIGIT^+^CD8 inhibitory T cells during MM progression. **a** Bar plot showing the proportions of T cell types of NBM (normal bone marrow, *n* = 67), MGUS (monoclonal gammopathy of unknown significance, *n* = 122), SMM (smoldering multiple myeloma, *n* = 122), and NDMM (newly diagnosed multiple, *n* = 704) from CIBERSORT analysis. Error bars represent mean ± standard error mean (SEM). *p* values for each cell type were calculated using Kruskal-Wallis with Dunn’s multiple comparisons test. b Correlation analysis of CD8 T cell (left, Pearson *r* = −0.308, *p* = 0.001.) and γδT cells (right, Pearson *r* = 0.219, *p* = 0.021) proportions with the time of progression in MGUS patients. **c** Violin plot showing the proportions of the CD8 T cells (left), γδT cells (middle), and naïve CD4 T cells (right) among high-risk (*n* = 35) and low-risk (*n* = 77) SMM patients from CIBERSORT analysis. Error bars represent mean ± standard error mean (SEM). *p* values were calculated using Mann–Whitney test. **d** Box plot showing T cell composition changes among different disease stages by CyTOF analysis. *p* values for each cell type were calculated using Kruskal-Wallis with Dunn’s multiple comparisons test. **e** Heatmap showing median intensity of indicated channels in each T cell types. *p* values for each cell type were calculated using Kruskal-Wallis with Dunn’s multiple comparisons test.

CIBERSORT analysis of the BM biopsy GEP data revealed a 53% increase in CD8^+^ T cells, a 63% decrease in naïve CD4^+^ T cells, and a 42% decrease in activated CD4^+^ memory T cells in NDMM compared to NBM (Fig. 5a). We observed that high proportions of CD8^+^ T cells while low proportions of γδ T cells correlated with short progression time in SMM patients (Fig. 5b). We observed low proportions of γδ T cells were also correlated with short progression time in MGUS patients (Supplementary Fig. 6). Consistently, high-risk SMM patients showed higher proportions of CD8^+^ T cells and lower proportions of naïve CD4^+^ T cells and γδ T cells (Fig. 5c). However, we did not find that T cell subpopulations were associated with OS or EFS in NDMM (Supplementary Fig. 7).

To investigate the phenotype and function of T cell populations, we analyzed the 5T cell MCs identified by FlowSOM analysis of CyTOF data, including a population of CCR7^+^CD45RA^-^ central memory T cells (Tcm), two CCR7^-^CD45RA^-^ effector memory T cells (Tem), and two CD57^+^ terminal differentiation T cells (Temra). We observed an increase of CD8^+^ Tem, CD8^+^ Temra, CD4^+^ Tem, and CD4^+^ Tcm proportion at the precursor stages (Fig. 5d). Functional marker analysis identified an increase in CD25 and FOXP3 intensity among CD4^+^ Tcm in NDMM and its precursor stages (Fig. 5e), suggesting CD4^+^ Tcm exhibit immune inhibition in the TME. We evaluated immune checkpoint (PD1, TIGIT, TIM3, and LAG3) markers during different disease stages. PD1 was elevated in CD4^+^ Temra, while TIGIT was elevated in CD8^+^ Temra and CD8^+^ Tem. Both PD1 and TIGIT showed increased levels during disease progression (Fig. 5e). These data revealed an accumulation of inhibitory T cells in the TME.

### *GZMB*^*+*^ cytotoxic T cell subsets are enriched and show individual specificity in the TME

**Fig. 6:**
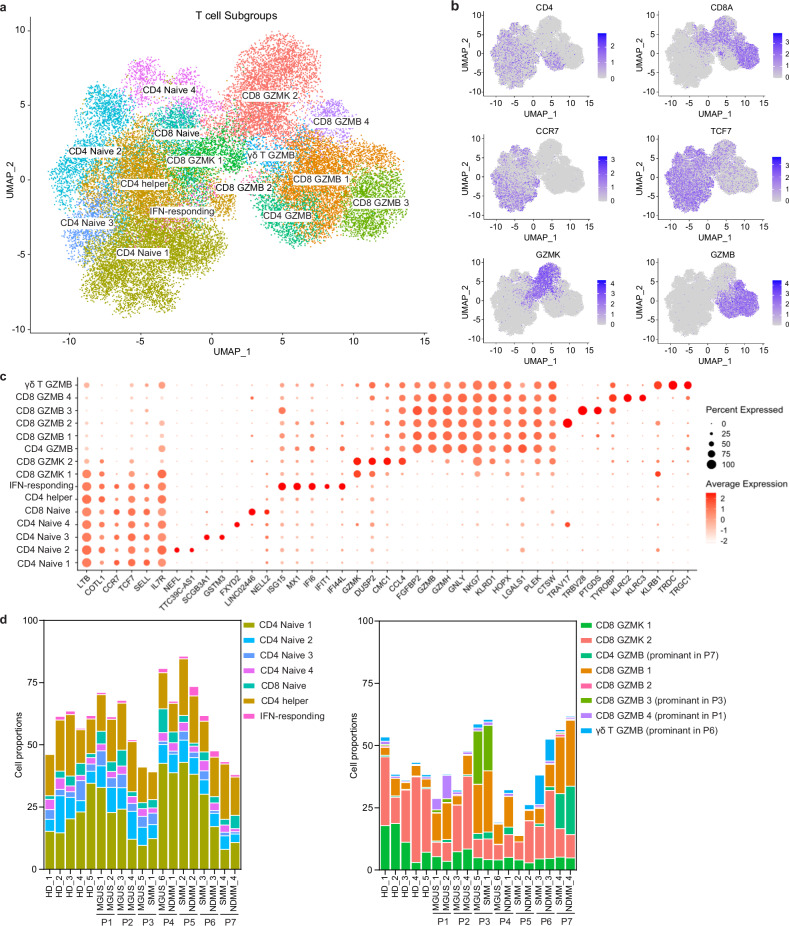
*GZMB*^*+*^ cytotoxic T cell subsets are enriched and show individual specificity in the TME. **a** UMAP plots of T cell subtypes. **b** Density map on the UMAP plot showing gene expression levels among T subtypes. **c** Dot plot showing marker gene expression of T cell subtypes. **d** stacked box plots showing the proportions of noncytotoxic T cell subtypes (left) and cytotoxic T cell subtypes (right). *p* values for each cell subtype were calculated using Benjamin–Hochberg corrected, two-sided Wilcoxon rank sum test.

Consistent with CIBERSORT and CyTOF analyses, scRNA-Seq also revealed an overall increase of total T cells in the TME (Fig. 3c, d). To delve deeper into the T cell subtypes and function, we performed additional subclustering at higher resolution using genes variably expressed among T cells, resulting in 15 subclusters (Fig. 6a). The non-cytotoxic subsets consist of four populations of *CCR7*^*+*^*TCF7*^*+*^ naïve CD4 subsets, one population of *CCR7*^*+*^*TCF7*^*+*^ naïve CD8 subset, one population of CD4 helper subset, and one IFN-responding CD4 subset. The cytotoxic subsets contain four populations of *GZMB*^*+*^ CD8 subsets, two *GZMK*^*+*^ CD8 subsets, one *GZMB*^*+*^ CD4 subset and one *GZMB*^*+*^ γδ T subset (Fig. 6a, b). The non-cytotoxic subsets were characterized by high expression of immune modulatory genes (*LTB, IL7R*). *GZMB*^*+*^ cytotoxic subsets expressed a great number of cytotoxic genes, such as *GZMB, GZMH, GNLY, NKG7, KLRD1*, while *GZMK*^*+*^ cytotoxic subsets expressed *GZMK* (Fig. 6c). Consistent with other reports, the proportion of the CD8 *GZMK*^*+*^ subset showed a trend of decrease while the CD8 *GZMB*^*+*^ subset showed a trend of increase in the TME (Fig. 6d). Interestingly, we observed that two CD8 *GZMB*^*+*^ subsets, one CD4 *GZMB*^*+*^ subset and one γδ T *GZMB*^*+*^ subset was predominantly derived from individual patients and were detected at both time points (Fig. 6d), suggesting that *GZMB*^*+*^ cytotoxic T cells may perform anti-tumor specific immune response in the TME.

We next examined the differential gene expression between TME and NBM. Similar to the findings with innate immune cells, we observed an upregulation of IFN responding genes and a downregulation of stress response genes in TME (Supplementary Fig. 8a, b). In addition, the expression levels of cytotoxic genes *LYZ, GZMK* were decreased while *PRF1, GZMH* were increased in the TME (Supplementary Fig. 8a, b). Interestingly, consistent with our previous finding that the *GZMB*^*+*^ cytotoxic subsets were specific in individual patients, the gene expression pattern of T cells also showed patient specificity instead of disease stage specificity (Supplementary Fig. 8b). Next, we examined the gene expression of T cell functional markers among T cell subsets. T cell activation markers *CD27, CD28*, and *CD69* showed high expression in the non-cytotoxic subsets. Genes encoding for MHC type II proteins, which have been reported to inhibit T cell proliferation and cytokine production, showed high expression in the cytotoxic subsets. The immune checkpoint gene *TIGIT* was expressed in cytotoxic CD8^+^ T cell subtypes. *CTLA4* was expressed in CD4 helper cells. *LAG3* was sporadically expressed in the cytotoxic subsets (Supplementary Fig. 8c). LAG3 had increased expression in patients at late stage compared with NBM in both the CD8 *GZMK*^*+*^ and CD8 *GZMB*^*+*^ subsets (Supplementary Fig. 8d). However, we did not see a difference in the expression of TIGIT and MHC class genes. Of note, TIGIT showed increased protein levels in our CyTOF analysis (Fig. 5f), thus TIGIT may be post-transcriptionally regulated in the TME. These data indicate that T cell subsets are altered in MM and its precursor stages, with a shift from *GZMK*^*+*^ CD8 to *GZMB*^*+*^ CD8 subsets. The *GZMB*^*+*^ CD8 subsets exhibit patient specificity in the TME, whether those patient-specific T cell subsets are related to specific tumor clones due to antigen activation needs further investigation.

### Composition alteration of MM subclones in paired samples shows patient-specific heterogeneity in progressed disease

**Fig. 7:**
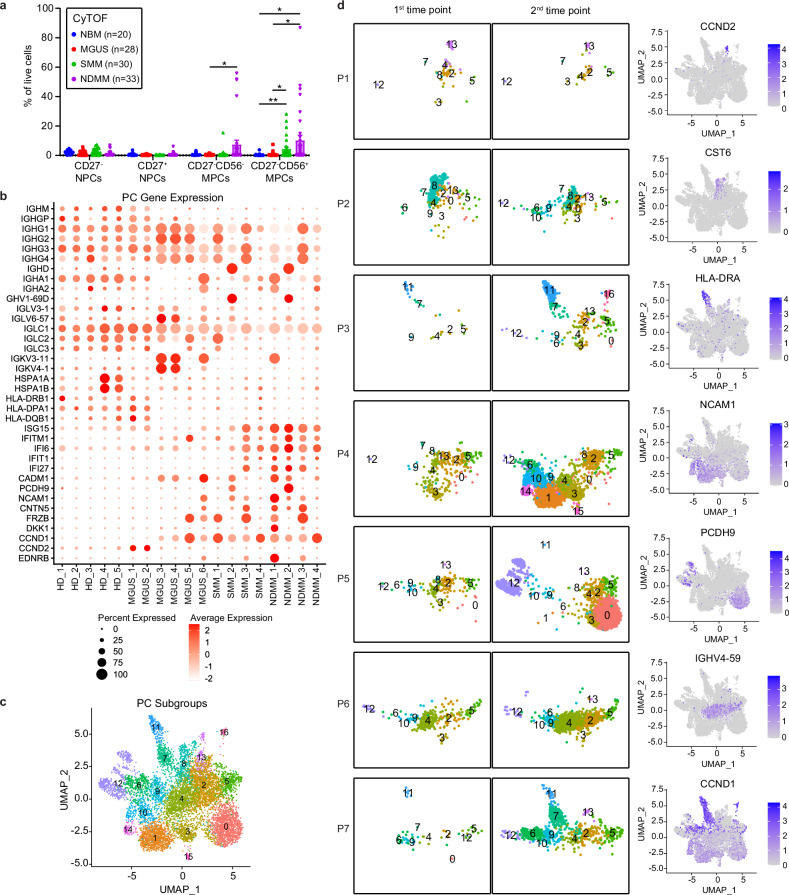
Composition alteration of MM clones in paired samples shows patient-specific heterogeneity in progressed disease. **a** Box plot showing plasma cell (PC) composition changes among different disease stages by CyTOF analysis. *p* values for each cell type were calculated using Kruskal–Wallis with Dunn’s multiple comparisons test. **b** Dot plot showing gene expression of plasma cells for individual patients and healthy donors. **c** UMAP plots of plasms cell subtypes. **d** UMAP plots showing plasma cell subclusters in paired samples (left). Density map on the UMAP plot showing representative marker gene expression among plasma cell subtypes (right).

Lastly, we studied the evolution of PC subclones during MM progression. FlowSOM analysis of CyTOF data identified two NPC MCs (CD27^-^ and CD27^+^) and two MPC MCs (CD27^-^CD56^-^ and CD27^-^CD56^+^). The proportion of CD27^-^CD56^-^ MPCs showed a 55% increase in SMM and a 10.6-fold increase in NDMM compared to NBM. The proportion of the CD27^-^CD56^+^ MPCs increased 10.0-fold in SMM and 24.3-fold in NDMM compared with NBM (Fig. 7a). It suggests that the CD27^-^CD56^+^ MPCs drive early tumor expansion. No significant difference was observed in the expression of complement inhibitory proteins CD55 and CD59, and immune checkpoint ligand PD-L1 of MPC MCs among NDMM, SMM, MGUS, and NBM (Supplementary Fig. 9), which may be due to the remarkable heterogeneity in the expression of these markers within the groups. The gene expression pattern of PCs cluster in scRNA-Seq analysis showed that PCs in healthy donors exhibited poly-PC signatures, but PCs in diseased patients showed an increase in percentage and intensity of genes encoding for either immunoglobulin heavy chain IgG, IgA or IgD, which were consistent with their pathological examination results (Fig. 7b, Supplementary Fig. 10, Supplementary Table 4). We also observed that PCs showed increased IFN-response genes in NDMM and part of the SMM patients (Fig. 7b). Moreover, we observed that genes related to cell division (*EDNRB, CCND1, CCND2*), Wnt signaling (*DKK1, FRZB*), and cell adhesion (*CNTN5, NCAM1* (encoding CD56), *PCDH9, CADM1*) were significantly increased in individual patients (Fig. 7b), indicating that MM and its precursor stages can be grouped into different subtypes based on their gene expression. Notably, many of those genes have been identified in CD138^+^ MM cell GEP analysis for the characterization of 7 MM groups [32]. Next, we subclustered plasma cells into 24 plasma cell subtypes based on their gene expression (Fig. 7c, Supplementary Fig. 11). Stable MGUS paired samples (P1, P2) showed few compositional alterations of the MM clone. Two of the five paired progression samples showed expansion of the dominant subclone (P3, P6); 3/5 Progressive paired samples showed alteration of dominant subclone (P4, P5, P7) (Fig. 7d, Supplementary Fig. 12). Whether the uncontrolled clonal expansion and composition alterations of MM cells in the paired progression samples can be explained by the lack of adaptation of cytotoxic T cells during disease progression needs biological examination.

## Discussion

Early intervention at MM precursor stages to prevent disease progression represents an attractive treatment option. However, it is challenging to accurately predict which MGUS and SMM patients will progress, making it essential to avoid treating those who may never develop MM. A more in-depth study of the mechanisms of MGUS and SMM progression requires comprehensive molecular characterization, including the changing microenvironment. In this study, we analyzed the immune cell compositional, functional, and transcriptional alterations in MM and its precursor stages by integrating whole BM biopsy-based CIBERSORT analysis with CyTOF and scRNA-Seq analysis in a large cohort of patients with long follow-up.

Analyzing whole BM biopsies of patients with MM and its precursor conditions identified a continued decrease of granulocyte proportions, especially the neutrophils and mast cells, from normal BM to MGUS, to SMM, and to MM. The very low-granulocyte phenotype was observed in MM patients with high-GEP-70 and ISS stage III and predicted an inferior survival outcome. A low-granulocyte phenotype was enriched in relapsed and refractory MM patients and was shown to add additional prognostic information to standard tumor-specific risk-stratification tools [27]. However, we noted that CIBERSORT analysis does not distinguish mature and immature neutrophils. A recent study using flow cytometry-based cell separation followed by scRNA-Seq analysis provided a more accurate analysis of neutrophil function in MM [33]. Neutrophil dysfunction has been found in MM, SMM, and MGUS patients evidenced by reduced phagocytosis and oxidative burst ability compared with healthy donors [34]. This likely explains why MM patients are prone to infections and suggests that the number of granulocytes within the BM could be used as an additional criterium for risk stratification of MM and prediction of MM progression. Moreover, we used CyTOF and scRNA-Seq of patient samples across all stages of MM to elucidate the surface protein markers and transcriptomic alterations within the immune microenvironment during disease progression. Immature granulocyte proportions within the BM immune microenvironment were decreased in diseased compared with normal BM, although it needs to be noted that BM aspirate samples lose significant numbers of mature granulocytes during Ficoll separation. Similar to what we found in mDCs and other cell types, the stress-response genes in immature granulocytes were suppressed in the TME. It suggests that even a limited number of tumor cells in the MM precursor stages can impair the generation and maturation of granulocytes. Based on these findings, adoptive neutrophil-based therapy may prevent MM progression.

Inhibitory immune checkpoints are upregulated not only in T cells but also in NK cells in the tumor microenvironment [35, 36]. Studies have shown increased TIGIT expression in CD8^+^ T cells [37] and NK cells [38] in MM during disease progression. Increased PD1 expression has been observed in CD8^+^ T cells at SMM and MM stages [39], as well as after BCMA-CAR-T cell treatment of relapsed MM [40]. Recent findings have shown that the generation of NK cells with PD1-based chimeric-switch receptors enhances and sustains potent anti-tumor activity in a PD-L1^+^ microenvironment [41]. However, immune checkpoint inhibition with monoclonal antibodies has failed in MM due to uncontrollable immune responses in combination therapies and the lack of efficacy of monotherapies in relapsed/refractory MM [42]. Thus, precise modulations of inhibitory immune checkpoints in NK and T cells are needed. Here, we observed increased TIM3 and TIGIT in cytotoxic NK cells beginning at MGUS and SMM stages. PD1 showed increased expression in CD4^+^ Temra, and TIGIT CD8^+^ Temra beginning at MGUS stage. Our findings suggest that specific inhibition of TIM3 and TIGIT in NK cells, inhibition of PD1 in CD4^+^ Temra, and inhibition of TIGIT in CD8^+^ Temra could be promising strategies to advance NK and T cell-based immunotherapies in MM and its precursor stages.

Examining BM immune cell gene expression alteration using scRNA-Seq techniques revealed loss of stress-response genes and elevation of IFN gene signaling among multiple innate and adaptive immune cell types. The enrichment of IFN-response genes has also been observed by others in MM [20, 43] and other hematological malignancies [44]. Our data appear to implicate IFN signaling in MM and that improving immune cell fitness by augmentation of the stress response pathways in MGUS and SMM stages may prevent disease progression. We also observed limited tumor surveillance and cytotoxic function of NK and CD8^+^ T cells. NK cells and CD8^+^ T cells showed common features with a shift from *GZMK*^*+*^ to *GZMB*^*+*^ cytotoxic phenotype in the diseased compared to normal BM and this was accompanied by increased expressions of inhibitory markers TIM3 and TIGIT, respectively. This is consistent with findings by other investigators suggesting impaired NK and T cell memory cells in the TME [20, 21], indicating that improving NK and T cell surveillance could be a potential therapeutic intervention to prevent MM progression.

Our analysis also investigated the normal plasma cells and MM cells in the BM across all stages of MM. CyTOF analysis showed two types of normal plasma cells, distinguished by either CD27^-^ and CD27^+^, and two types of malignant plasma cells, distinguished by either CD27^-^CD56^-^ or CD27^-^CD56^+^. We found an increased CD27^-^CD56^-^ MPCs were mostly in the NDMM group, while CD27^-^CD56^+^ MPCs were increased in both NDMM and SMM groups, suggesting that the CD27^-^CD56^+^ MPC subtype drives early tumor expansion. This could be related to CD56-mediated cellular adhesion and signaling in MM [45]. The rapid expansion of CD27^-^CD56^-^ MPCs from its precursor stages also appears to indicate that CD27^-^CD56^-^ MPCs are more aggressive. This is consistent with earlier findings that the absence of CD56 expression in MM is correlated with plasma cell leukemia, an aggressive form of MM [46]. Indeed, MM patients who are CD56^-^ have a significantly inferior overall survival than the CD56^+^ patients [47]. scRNA-Seq analysis showed heterogeneity of MM subclones based on their gene expression. We showed a loss of poly-PC signature in MGUS, SMM, and MM, while the immunoglobulin signatures remained consistent within the same patients. This is consistent with a previous GEP study in SMM patients [48], indicating that poly PC scores could be used to predict the time of MM progression.

## Materials and methods

### Ethics approval and consent to participate

All methods were performed in accordance with the relevant guidelines and regulations. All patients gave written informed consent and collection was approved by the University of Arkansas for Medical Sciences (UAMS) review boards (IRB #261820). BM biopsy samples were taken from precursor stages of MM and NDMM patients between 2004 and 2019 and subsequent for gene expression using Afflix. BM aspirates from MM patients were collected at the time of diagnosis and were diluted to 25 mL with PBS and overlaid on lymphocyte separation media. Following centrifugation, the collected buffy coat was washed with PBS before being frozen and stored in liquid nitrogen. Live frozen BM aspirate samples from precursor stages of MM and NDMM collected between 2017 and 2023 were taken for CyTOF and scRNA-Seq. MM% derived in our CyTOF and scRNA-Seq analysis were compared with the pathology MM%, outliers were excluded from the study, indicating hemodilution of the samples [49]. Healthy BM samples were obtained from donors without tumors. Age, gender, and clinical and laboratory characteristics of all participants are shown in Supplementary Tables 1, 2, 4.

### CIBERSORT analysis—deconvolution cell types of the BM biopsy

To deconvolve the cellular content of the BM, we implemented a pre-modified 27-myeloma genome signature matrix (MGSM27) [27] that involving a 22-leukocyte signature matrix [26] with an additional 4 BM cell types. The deconvolution method can be accessed on an online platform. The proportion of the 27 cell types in the BM was exported via importing gene expression profiling data of the BM biopsy.

### Mass cytometry (CyTOF) sample preparation and staining

Cryopreserved BM mononuclear cells were rapidly thawed, washed once with pre-warmed RPMI medium containing benzonase, and incubated at 37 °C 5% CO_2_ incubator for 1–2 h. Cells were then washed and resuspended in Cell staining buffer (CSB), 5 µL human TruStain FcXTM was added for 10 min on ice to block Fc receptor binding. Then metal-conjugated surface antibodies were added to the sample for another 30 min incubation on ice. Then cells were washed with CSB and stained with 5 mM cisplatin to label dead cells before incubating with Fix & Perm Buffer (Fluidigm) for 45 min at 4 °C. Intracellular antibodies were then added and incubated for another 30 min at RT. After washing with Perm buffer, the stained cells were incubated with 2% PFA with Iridium intercalator overnight at 4 °C. All antibodies were titrated to optimal staining concentrations using BMMC samples from patients.

### Mass cytometry (CyTOF) data acquisition

A Helios mass cytometer (Fluidigm) was used for data acquisition. Plasma start-up, background check, and tuning with metals (133Cs, 139La, 155Gd, 159Tb, 169Tm, 193Ir) were completed successfully according to the manufacturer instructions before running samples. Stained samples were washed twice with CSB and twice with ultrapure water. Prior to acquisition, cells were resuspended in water containing 10% EQ Four Element Calibration Beads (Fluidigm) at a concentration 0.5 million cells/mL and filtered through a 35 µm cell strainer. The samples were acquired at an acquisition rate of 300 - 500 events per second. A total of 0.5 million cells for each sample were collected. FCS files were normalized using calibration beads using CyTOF software 6.7 (Fluidigm).

### Mass cytometry (CyTOF) data analysis

Normalized files were uploaded to an online analyzer tool, Cytobank (Beckman Coulter). Beads, debris, doublets, and dead cells were excluded from the events using Gaussian parameters cleanup strategy [50]. Cleaned single-cell data were gated for cell populations according to marker expression and subsequently used for high-dimensional analyses as discussed in the results section. Cell populations were characterized by the expression of the cluster of differentiation markers, clustered by the FlowSOM algorithm, and visualized with vi-SNE plots. For these analyses, each sample contributed the same number of gated cells to minimize any bias. Bar plots and heatmaps were generated using the GraphPad Prism software (GraphPad, San Diego, CA, USA).

### scRNA-Seq sample preparation

Cryopreserved BM mononuclear cells were rapidly thawed, washed twice with PBS, stained with cell viability dyes and counted using a fluorescence microscope. Samples with viability higher than 95% were processed for 10x genomics scRNA-Seq. Approximately 10,000 cells were targeted, and single-cell emulsions were generated with the Chromium Controller (10x Genomics, Pleasanton, CA, USA) using the Chromium Next GEM Chip G (Part Number 1000120, 10x Genomics) with the Chromium NextGEM Single Cell 3’ v3.1 kit (Part Number 1000121, 10x Genomics) following the standard protocol. Libraries were assessed for mass concentration using the Qubit 1X dsDNA HS Assay Kit (catalog # Q33231; Invitrogen, Waltham MA, USA) and the Qubit 4 fluorometer (catalog # Q33238; Invitrogen, Waltham MA, USA). Library fragment size was assessed with the High Sensitivity NGS Fragment Analysis Kit (catalog # DNF-474-0500; Agilent, Santa Clara CA, USA) on the Fragment Analyzer System (catalog # M5310AA; Agilent, Santa Clara CA, USA). Libraries were functionally validated with the KAPA Universal Library Quantification Kit (catalog # 07960140001; Roche, Basel CH). Initial low-pass “surveillance” sequencing was performed on an Illumina NovaSeq SP 100-cycle flow cell and data were assessed using the Cell Ranger Count output.

### scRNA-Seq data analysis

Raw scRNA-Seq data were preprocessed using the Cell Ranger analysis pipelines (10x Genomics) version 6 with reference genome of the human genome (GRCh38) to demultiplex for cell and transcript and generate count table. The count table was loaded into R through the Seurat version 4 package for further analysis. Cells that have gene numbers less than 200, greater than 7,000, and more than 10% of unique molecular identifiers stemming from mitochondrial genes were discarded from the analysis. For individual samples, a principal component analysis (PCA) was performed on significantly variable genes for remaining high-quality cells. Results of individual samples were used for data integration across samples using the reciprocal PCA method to minimize technical differences between samples. The integration results were employed as input for clustering using the Louvain algorithm with multilevel refinement and the Uniform Manifold Approximation and Projection for Dimension Reduction (UMAP). Cell types were assigned using gene markers listed in Supplementary Table 5. Erythroid clusters were excluded from further analyses. To analyze the heterogeneity of indicated cell clusters, we selected each cluster and performed subcluster analysis using Seurat’s FindClusters function.

### Statistical analysis

Overall survival (OS) and event-free survival (EFS) curves were evaluated using the Kaplan–Meier method and compared using the log-rank test. The Wilcoxon rank sum test or Mann–Whitney test was performed on two groups and Kruskal–Wallis test was performed on three or more groups that do not have equal standard deviations (SD). Error bars represent mean ± standard error of mean (SEM) unless otherwise indicated. A *p*-value < 0.05 was considered statistically significant.

## Supplementary information

Supplemental Figures 1-12 and Supplemental Tables 1-4

Supplemental Table 5

## Data Availability

The micro-array data and scRNA-Seq data have been deposited in the Gene Expression Omnibus (GEO) database under accession code GSE276561 and GSE271107, respectively. The Raw files of CyTOF data is available upon request to the corresponding author.

## Code availability

Codes for survival analysis and scRNA-Seq analysis used in this paper have been deposited to GitHub (https://github.com/visanuwan/MM_progression_survival_singlecell).

## References

[1] Kyle RA, Rajkumar SV. Multiple myeloma. N Engl J Med. 2004;351:1860–73. 15509819 10.1056/NEJMra041875

[2] Kyle RA, Therneau TM, Rajkumar SV, Larson DR, Plevak MF, Offord JR, et al. Prevalence of monoclonal gammopathy of undetermined significance. N Engl J Med. 2006;354:1362–9. 16571879 10.1056/NEJMoa054494

[3] Kyle RA, Remstein ED, Therneau TM, Dispenzieri A, Kurtin PJ, Hodnefield JM, et al. Clinical course and prognosis of smoldering (asymptomatic) multiple myeloma. N Engl J Med. 2007;356:2582–90. 17582068 10.1056/NEJMoa070389

[4] Kyle RA, Rajkumar SV. Management of monoclonal gammopathy of undetermined significance (MGUS) and smoldering multiple myeloma (SMM). Oncology (Williston Park). 2011;25:578–86. 21888255 PMC3923465

[5] Maciocia N, Wechalekar A, Yong K. Monoclonal gammopathy of undetermined significance (MGUS) and smoldering myeloma (SMM): a practical guide to management. Hematol Oncol. 2017;35:432–9. 27804161 10.1002/hon.2345

[6] Dhodapkar MV, Sexton R, Waheed S, Usmani S, Papanikolaou X, Nair B, et al. Clinical, genomic, and imaging predictors of myeloma progression from asymptomatic monoclonal gammopathies (SWOG S0120). Blood. 2014;123:78–85. 24144643 10.1182/blood-2013-07-515239 PMC3879908

[7] Dutta AK, Fink JL, Grady JP, Morgan GJ, Mullighan CG, To LB, et al. Subclonal evolution in disease progression from MGUS/SMM to multiple myeloma is characterised by clonal stability. Leukemia. 2019;33:457–68. 30046162 10.1038/s41375-018-0206-x PMC6365384

[8] Oliva S, De Paoli L, Ruggeri M, Caltagirone S, Troia R, Oddolo D, et al. A longitudinal analysis of chromosomal abnormalities in disease progression from MGUS/SMM to newly diagnosed and relapsed multiple myeloma. Ann Hematol. 2021;100:437–43. 33392702 10.1007/s00277-020-04384-w

[9] Zingone A, Kuehl WM. Pathogenesis of monoclonal gammopathy of undetermined significance and progression to multiple myeloma. Semin Hematol. 2011;48:4–12. 21232653 10.1053/j.seminhematol.2010.11.003 PMC3040450

[10] Nakamura K, Smyth MJ, Martinet L. Cancer immunoediting and immune dysregulation in multiple myeloma. Blood. 2020;136:2731–40. 32645135 10.1182/blood.2020006540

[11] Dhodapkar KM, Cohen AD, Kaushal A, Garfall AL, Manalo RJ, Carr AR, et al. Changes in bone marrow tumor and immune cells correlate with durability of remissions following BCMA CAR T therapy in myeloma. Blood Cancer Discov. 2022;3:490–501. 36026513 10.1158/2643-3230.BCD-22-0018 PMC9627239

[12] Gandhi UH, Cornell RF, Lakshman A, Gahvari ZJ, McGehee E, Jagosky MH, et al. Outcomes of patients with multiple myeloma refractory to CD38-targeted monoclonal antibody therapy. Leukemia. 2019;33:2266–75. 30858549 10.1038/s41375-019-0435-7 PMC6820050

[13] van de Donk N, Richardson PG, Malavasi F. CD38 antibodies in multiple myeloma: back to the future. Blood. 2018;131:13–29. 29118010 10.1182/blood-2017-06-740944

[14] Dalla Palma B, Marchica V, Catarozzo MT, Giuliani N, Accardi F. Monoclonal and bispecific anti-BCMA antibodies in multiple myeloma. J Clin Med. 2020;9:3022. 10.3390/jcm9093022 PMC7565079 32961764

[15] Al Hadidi S, Heslop HE, Brenner MK, Suzuki M. Bispecific antibodies and autologous chimeric antigen receptor T cell therapies for treatment of hematological malignancies. Mol Ther. 2024;32:2444–60. 10.1016/j.ymthe.2024.05.039 PMC11405165 38822527

[16] Hipp S, Tai YT, Blanset D, Deegen P, Wahl J, Thomas O, et al. A novel BCMA/CD3 bispecific T-cell engager for the treatment of multiple myeloma induces selective lysis in vitro and in vivo. Leukemia. 2017;31:2278. 28751764 10.1038/leu.2017.219

[17] Cho SF, Lin L, Xing L, Li Y, Wen K, Yu T, et al. The immunomodulatory drugs lenalidomide and pomalidomide enhance the potency of AMG 701 in multiple myeloma preclinical models. Blood Adv. 2020;4:4195–207. 32898244 10.1182/bloodadvances.2020002524 PMC7479960

[18] Seckinger A, Delgado JA, Moser S, Moreno L, Neuber B, Grab A, et al. Target expression, generation, preclinical activity, and pharmacokinetics of the BCMA-T cell bispecific antibody EM801 for multiple myeloma treatment. Cancer Cell. 2017;31:396–410. 28262554 10.1016/j.ccell.2017.02.002

[19] Yao L, Jayasinghe RG, Lee BH, Bhasin SS, Pilcher W, Doxie DB, et al. Comprehensive characterization of the multiple myeloma immune microenvironment using integrated scRNA-seq, CyTOF, and CITE-seq analysis. Cancer Res Commun. 2022;2:1255–65. 36969740 10.1158/2767-9764.CRC-22-0022 PMC10035369

[20] Zavidij O, Haradhvala NJ, Mouhieddine TH, Sklavenitis-Pistofidis R, Cai S, Reidy M, et al. Single-cell RNA sequencing reveals compromised immune microenvironment in precursor stages of multiple myeloma. Nat Cancer. 2020;1:493–506. 33409501 10.1038/s43018-020-0053-3 PMC7785110

[21] Schinke C, Poos AM, Bauer M, John L, Johnson S, Deshpande S, et al. Characterizing the role of the immune microenvironment in multiple myeloma progression at a single-cell level. Blood Adv. 2022;6:5873–83. 35977111 10.1182/bloodadvances.2022007217 PMC9647426

[22] Ho M, Patel A, Goh CY, Moscvin M, Zhang L, Bianchi G. Changing paradigms in diagnosis and treatment of monoclonal gammopathy of undetermined significance (MGUS) and smoldering multiple myeloma (SMM). Leukemia. 2020;34:3111–25. 33046818 10.1038/s41375-020-01051-x

[23] Korde N, Kristinsson SY, Landgren O. Monoclonal gammopathy of undetermined significance (MGUS) and smoldering multiple myeloma (SMM): novel biological insights and development of early treatment strategies. Blood. 2011;117:5573–81. 21441462 10.1182/blood-2011-01-270140 PMC3316455

[24] Nooka AK, Wang ML, Yee AJ, Kaufman JL, Bae J, Peterkin D, et al. Assessment of safety and immunogenicity of PVX-410 vaccine with or without lenalidomide in patients with smoldering multiple myeloma: a nonrandomized clinical trial. JAMA Oncol. 2018;4:e183267. 30128502 10.1001/jamaoncol.2018.3267 PMC6440721

[25] Li Y. DNA vaccines against GPRC5D in myeloma. Blood. 2023;142:5776.

[26] Newman AM, Liu CL, Green MR, Gentles AJ, Feng W, Xu Y, et al. Robust enumeration of cell subsets from tissue expression profiles. Nat Methods. 2015;12:453–7. 25822800 10.1038/nmeth.3337 PMC4739640

[27] Danziger SA, McConnell M, Gockley J, Young MH, Rosenthal A, Schmitz F, et al. Bone marrow microenvironments that contribute to patient outcomes in newly diagnosed multiple myeloma: a cohort study of patients in the Total Therapy clinical trials. PLoS Med. 2020;17:e1003323. 33147277 10.1371/journal.pmed.1003323 PMC7641353

[28] Danziger SA, Gibbs DL, Shmulevich I, McConnell M, Trotter MWB, Schmitz F, et al. ADAPTS: Automated deconvolution augmentation of profiles for tissue specific cells. PLoS ONE. 2019;14:e0224693. 31743345 10.1371/journal.pone.0224693 PMC6863530

[29] Van Gassen S, Callebaut B, Van Helden MJ, Lambrecht BN, Demeester P, Dhaene T, et al. FlowSOM: Using self-organizing maps for visualization and interpretation of cytometry data. Cytometry A. 2015;87:636–45. 25573116 10.1002/cyto.a.22625

[30] Vogt L, Schmitz N, Kurrer MO, Bauer M, Hinton HI, Behnke S, et al. VSIG4, a B7 family-related protein, is a negative regulator of T cell activation. J Clin Invest. 2006;116:2817–26. 17016562 10.1172/JCI25673 PMC1578631

[31] Nielsen CM, White MJ, Goodier MR, Riley EM. Functional significance of CD57 expression on human NK cells and relevance to disease. Front Immunol. 2013;4:422. 24367364 10.3389/fimmu.2013.00422 PMC3856678

[32] Zhan F, Huang Y, Colla S, Stewart JP, Hanamura I, Gupta S, et al. The molecular classification of multiple myeloma. Blood. 2006;108:2020–8. 16728703 10.1182/blood-2005-11-013458 PMC1895543

[33] de Jong MME, Fokkema C, Papazian N, Czeti A, Appelman MK, Vermeulen M, et al. An IL-1beta-driven neutrophil-stromal cell axis fosters a BAFF-rich protumor microenvironment in individuals with multiple myeloma. Nat Immunol. 2024;25:820–33. 38600356 10.1038/s41590-024-01808-x

[34] Askman S, Westerlund J, Pettersson A, Hellmark T, Johansson A, Wichert S, et al. Decreased neutrophil function in newly diagnosed multiple myeloma patients is restored with lenalidomide therapy. Eur J Haematol. 2024;113:72–81. 10.1111/ejh.14200 38553844

[35] Martinez-Perez A, Aguilar-Garcia C, Gonzalez S. The emerging role of NK cells in immune checkpoint blockade. Cancers (Basel). 2022;14:6005. 10.3390/cancers14236005 PMC9736655 36497486

[36] Liu Z, Xu X, Liu H, Zhao X, Yang C, Fu R. Immune checkpoint inhibitors for multiple myeloma immunotherapy. Exp Hematol Oncol. 2023;12:99. 38017516 10.1186/s40164-023-00456-5 PMC10685608

[37] Guillerey C, Harjunpaa H, Carrie N, Kassem S, Teo T, Miles K, et al. TIGIT immune checkpoint blockade restores CD8(+) T-cell immunity against multiple myeloma. Blood. 2018;132:1689–94. 29986909 10.1182/blood-2018-01-825265

[38] Liu ZY, Deng L, Jia Y, Liu H, Ding K, Wang W, et al. CD155/TIGIT signalling plays a vital role in the regulation of bone marrow mesenchymal stem cell-induced natural killer-cell exhaustion in multiple myeloma. Clin Transl Med. 2022;12:e861. 35858240 10.1002/ctm2.861 PMC9299950

[39] Costa F, Vescovini R, Marchica V, Storti P, Notarfranchi L, Dalla Palma B, et al. PD-L1/PD-1 pattern of expression within the bone marrow immune microenvironment in smoldering myeloma and active multiple myeloma patients. Front Immunol. 2020;11:613007. 33488620 10.3389/fimmu.2020.613007 PMC7820813

[40] Rade M, Grieb N, Weiss R, Sia J, Fischer L, Born P, et al. Single-cell multiomic dissection of response and resistance to chimeric antigen receptor T cells against BCMA in relapsed multiple myeloma. Nat Cancer. 2024;5:1318–33. 10.1038/s43018-024-00763-8 38641734

[41] Susek KH, Schwietzer YA, Karvouni M, Gilljam M, Keszei M, Hussain A, et al. Generation of NK cells with chimeric-switch receptors to overcome PD1-mediated inhibition in cancer immunotherapy. Cancer Immunol Immunother. 2023;72:1153–67. 36355079 10.1007/s00262-022-03317-y PMC10110653

[42] Jelinek T, Paiva B, Hajek R. Update on PD-1/PD-L1 inhibitors in multiple myeloma. Front Immunol. 2018;9:2431. 30505301 10.3389/fimmu.2018.02431 PMC6250817

[43] Boiarsky R, Haradhvala NJ, Alberge JB, Sklavenitis-Pistofidis R, Mouhieddine TH, Zavidij O, et al. Single cell characterization of myeloma and its precursor conditions reveals transcriptional signatures of early tumorigenesis. Nat Commun. 2022;13:7040. 36396631 10.1038/s41467-022-33944-z PMC9672303

[44] Dufva O, Polonen P, Bruck O, Keranen MAI, Klievink J, Mehtonen J, et al. Immunogenomic landscape of hematological malignancies. Cancer Cell. 2020;38:380–399 e313. 32649887 10.1016/j.ccell.2020.06.002

[45] Cottini F, Rodriguez J, Hughes T, Sharma N, Guo L, Lozanski G, et al. Redefining CD56 as a biomarker and therapeutic target in multiple myeloma. Mol Cancer Res. 2022;20:1083–95. 35380709 10.1158/1541-7786.MCR-21-0828

[46] Pellat-Deceunynck C, Barille S, Jego G, Puthier D, Robillard N, Pineau D, et al. The absence of CD56 (NCAM) on malignant plasma cells is a hallmark of plasma cell leukemia and of a special subset of multiple myeloma. Leukemia. 1998;12:1977–82. 9844928 10.1038/sj.leu.2401211

[47] Sahara N, Takeshita A, Shigeno K, Fujisawa S, Takeshita K, Naito K, et al. Clinicopathological and prognostic characteristics of CD56-negative multiple myeloma. Br J Haematol. 2002;117:882–5. 12060125 10.1046/j.1365-2141.2002.03513.x

[48] Khan R, Dhodapkar M, Rosenthal A, Heuck C, Papanikolaou X, Qu P, et al. Four genes predict high risk of progression from smoldering to symptomatic multiple myeloma (SWOG S0120). Haematologica. 2015;100:1214–21. 26022710 10.3324/haematol.2015.124651 PMC4800692

[49] Lee N, Moon SY, Lee JH, Park HK, Kong SY, Bang SM, et al. Discrepancies between the percentage of plasma cells in bone marrow aspiration and BM biopsy: Impact on the revised IMWG diagnostic criteria of multiple myeloma. Blood Cancer J. 2017;7:e530. 28211888 10.1038/bcj.2017.14 PMC5386332

[50] Bagwell CB, Inokuma M, Hunsberger B, Herbert D, Bray C, Hill B, et al. Automated data cleanup for mass cytometry. Cytometry A. 2020;97:184–98. 31737997 10.1002/cyto.a.23926

