# Supplementary material for: Multi-omics reveal immune microenvironment alterations in multiple myeloma and its precursor stages
Source: Blood Cancer J. 2024 Nov 6;14(1):194. doi: 10.1038/s41408-024-01172-x (PMC11541562; doi:10.1038/s41408-024-01172-x)
Supplement: Supplementary file 1 — Supplemental Figures 1-12 and Supplemental Tables 1-4 [file 41408_2024_1172_MOESM1_ESM.pdf]

Supplementary Information for the article

Multi-omics reveal immune microenvironment alterations in multiple myeloma and its precursor stages

Authors

Yan Cheng [1,#], Fumou Sun [1,#], Daisy V. Alapat [2], Visanu Wanchai [1], David Mery [1], Eric R Siegel [3], Hongwei Xu [1], Sarah Johnson [1], Wancheng Guo [1], Clyde Bailey [1], Cody Ashby [4], Michael Anton Bauer [4], Samer Al Hadidi [1], Carolina Schinke [1], Sharmilan Thanendrarajan [1], Maurizio Zangari [1], Frits van Rhee [1], Guido Tricot [1], John D Shaughnessy Jr [1,*], Fenghuang Zhan [1,*]

Affiliations

[1] Myeloma Center, Winthrop P. Rockefeller Institute, Department of Internal Medicine, University of Arkansas for Medical Sciences, Little Rock, AR 72205, USA.

[2] Department of Pathology, College of Medicine, University of Arkansas for Medical Sciences, Little Rock, AR 72205, USA.

[3] Department of Biostatistics, University of Arkansas for Medical Sciences, Little Rock, AR 72205, USA.

[4] Department of Biomedical Informatics, College of Medicine, University of Arkansas for Medical Sciences, Little Rock, AR 72205, USA.

[#]These authors contributed equally.

*Correspondence: Fzhan@uams.edu; JDShaughnessy@uams.edu

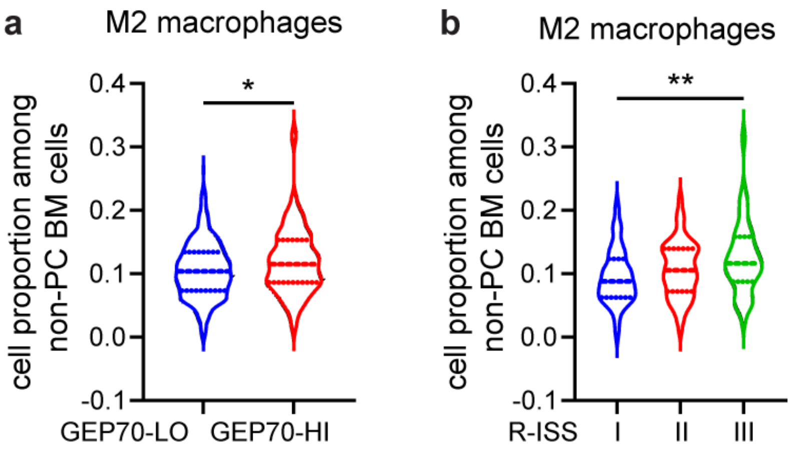

Supplemental Figure 1. CIBERSORT analysis revealed high levels of M2 macrophages correlate with high-risk disease in MM.

a. Violin plot showing the proportions of M2 macrophages in MM patients with GEP70-Low (n=598) and GEP70-High (n=105) (GEP70 cut off 0.66). *p* values were calculated using Mann-Whitney test.

b. Violin plot showing the proportions of M2 macrophages in MM patients in R-ISS stages I (n=88), II (n=193), and III (n=42) (right). *p* values for each cell type were calculated using Kruskal-Wallis with Dunn's multiple comparisons test.

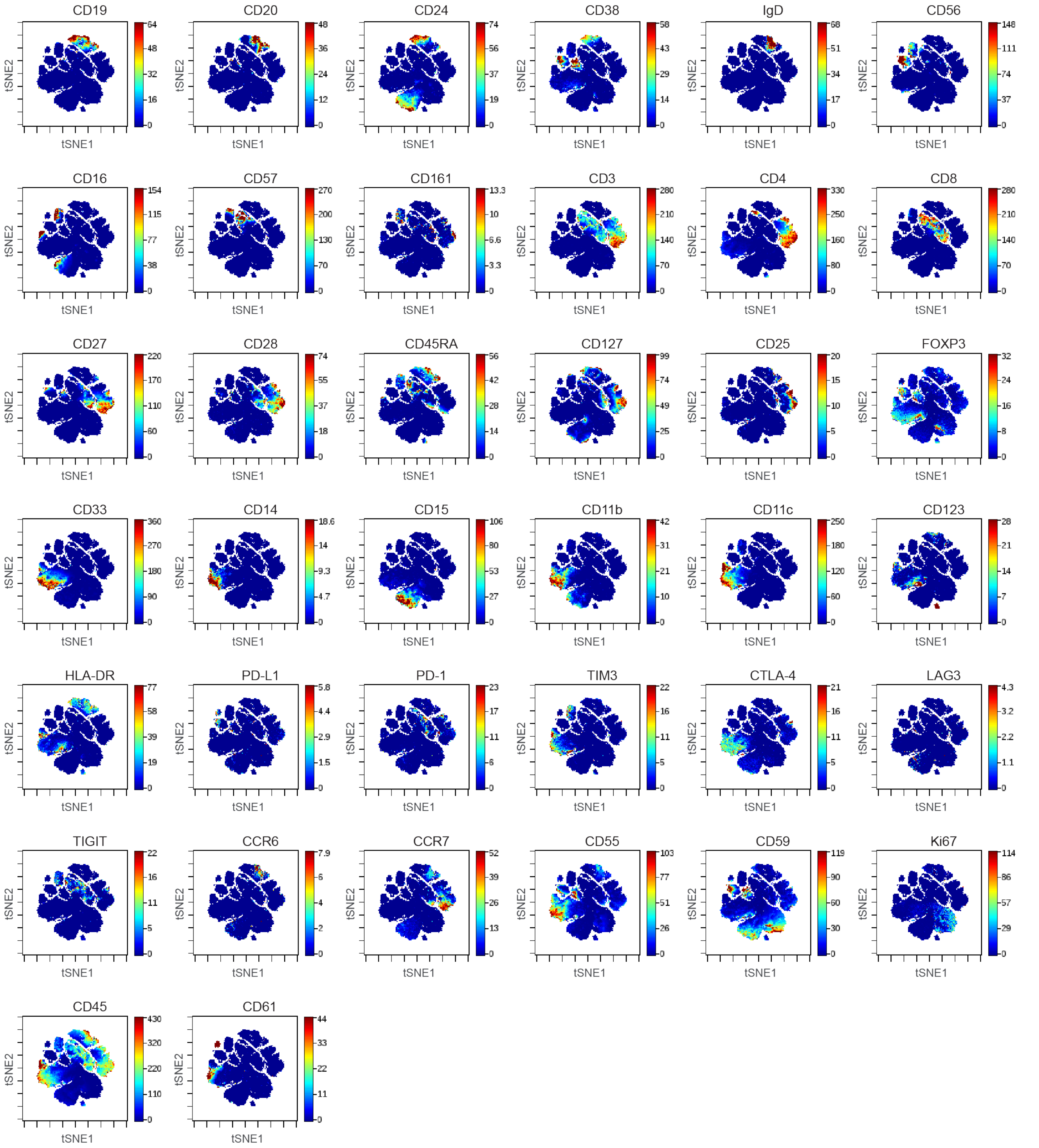

Supplemental Figure 2. The 38 channel of marker expression in viSNE plot of CyTOF analysis.

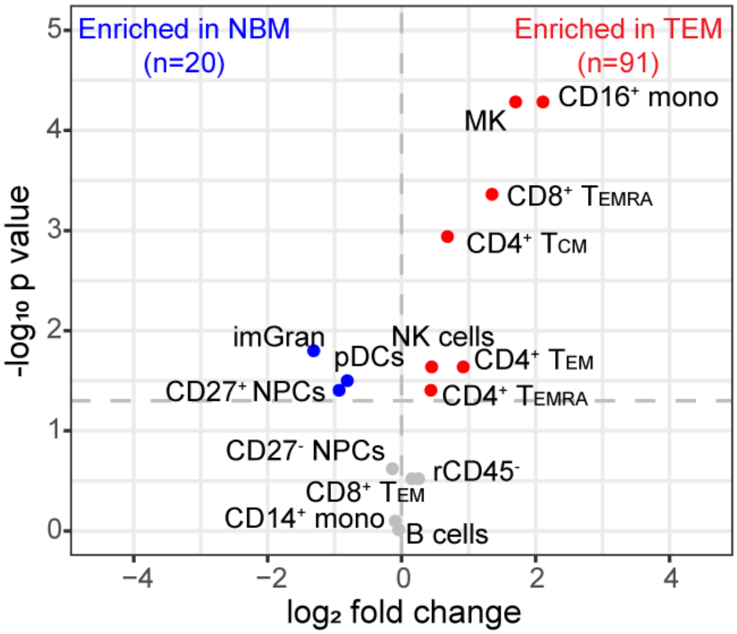

Supplemental Figure 3. Volcano plot showing metacluster (MC) changes between normal bone marrow (NBM) and tumor microenvironment (TME) after excluding the two malignant plasma cell (MPC) MCs in CyTOF analysis. For each cell type the log fold change in mean cell fraction between tumor and normal samples, with -log10 Benjamin-Hochberg-corrected, two-sided Wilcoxon rank-sum p values on the y-axis is shown.

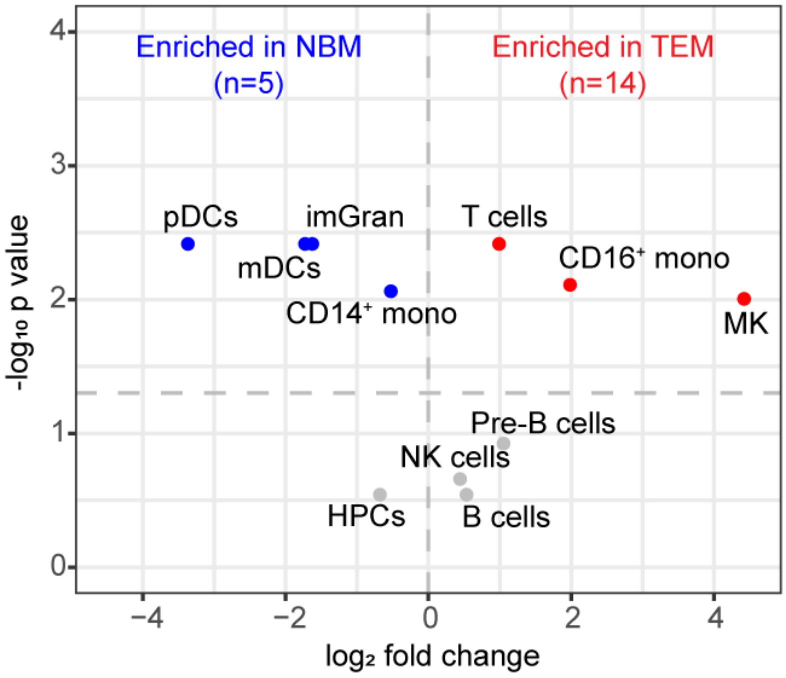

Supplemental Figure 4. Volcano plot showing cell cluster changes between normal bone marrow (NBM) and tumor microenvironment (TME) after excluding the Plasma Cell cluster in scRNA-Seq analysis. For each cell type the log fold change in mean cell fraction between tumor and normal samples, with -log10 Benjamin-Hochberg-corrected, two-sided Wilcoxon rank-sum p values on the y-axis is shown.

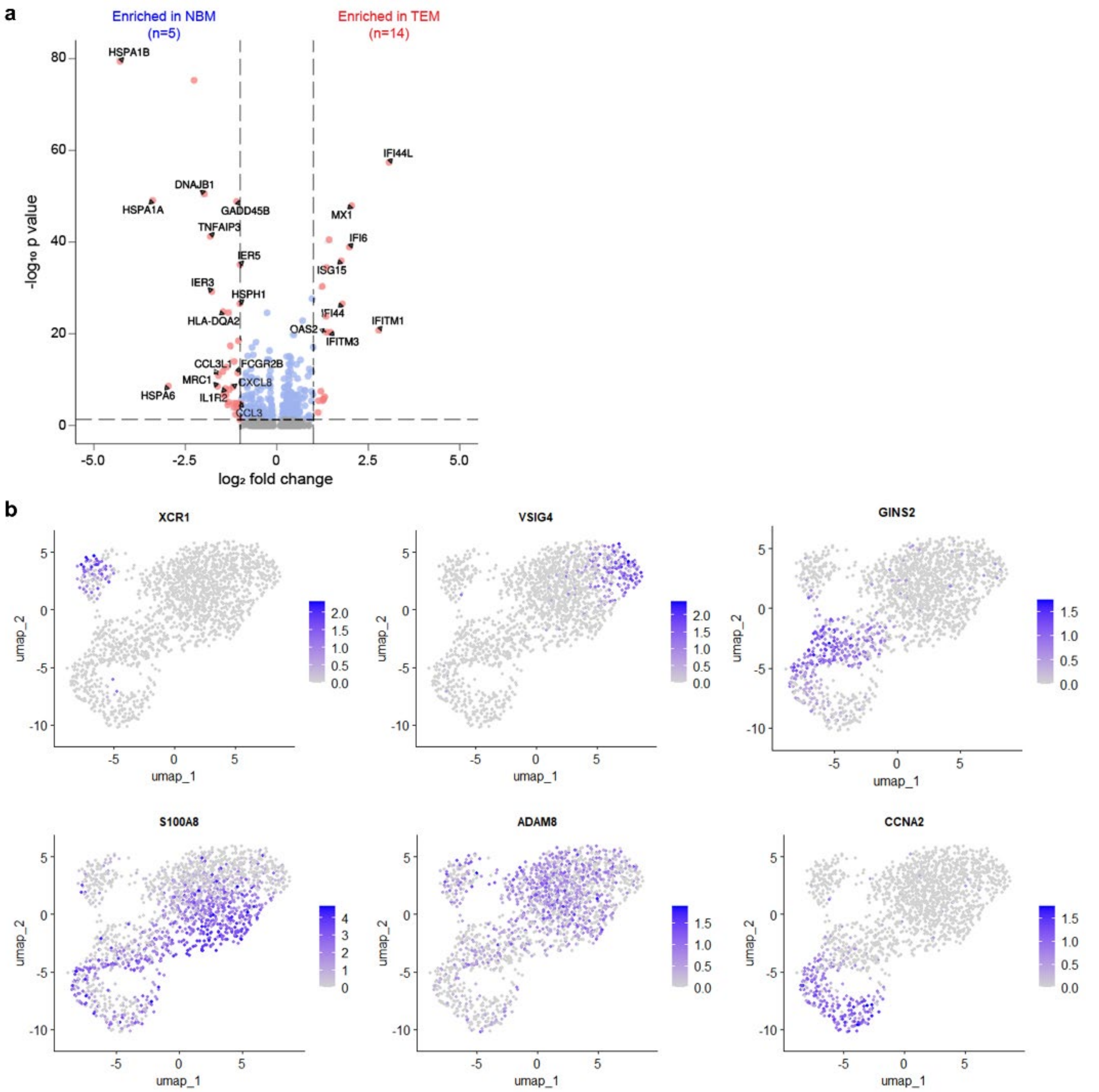

Supplemental Figure 5. The proportion of *VSIG4*⁺ mDCs subset decreased in the TME.

a. Volcano plot showing differential gene expression in myeloid dendritic cells between normal bone marrow (NBM) and tumor microenvironment (TME).

b. Density map on the UMAP plot showing indicated gene expression levels among mDC subtypes.

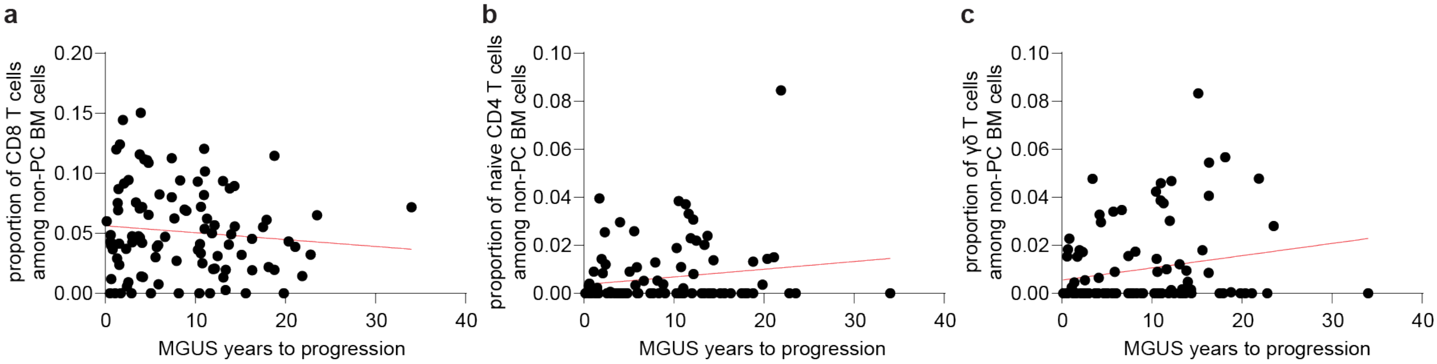

Supplemental Figure 6. Correlation analysis of T cell subset proportions in CIBERSORT analysis with the time of progression in MGUS patients.

a. Correlation analysis of $CD8^+$ T cell proportions with the time of progression in MGUS patients. Pearson r=-0.105, p>0.05.

b. Correlation analysis of naïve $CD4^+$ T cell proportions with the time of progression in MGUS patients. Pearson r=0.169, p>0.05.

c. Correlation analysis of γδ T cell proportions with the time of progression in MGUS patients. Pearson r=0.203, p=0.043.

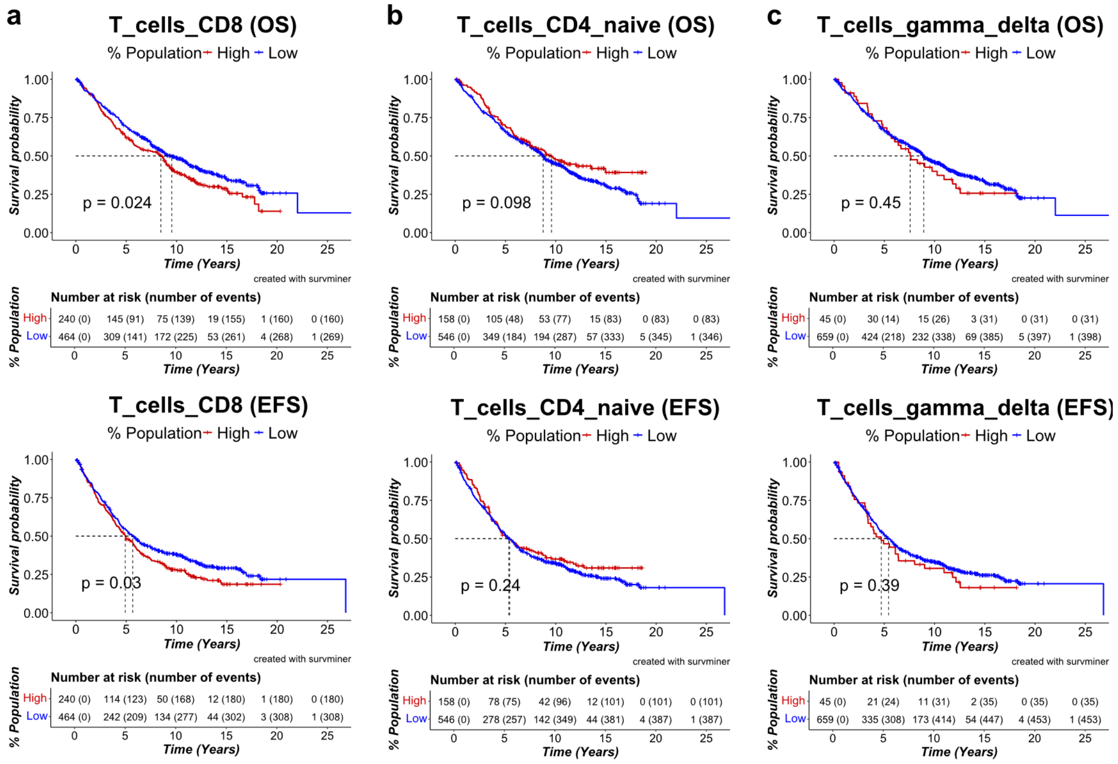

Supplemental Figure 7. T cell subset proportions in CIBERSORT analysis had no correlation with the outcomes of MM patients.

a. Kaplan-Meier analyses of overall survival (OS, left) and event-free survival (EFS, right) in MM patients with high and low $CD8^+$ T cells. Hazard ratios OS=0.798, *p*=0.02; Hazard ratios EFS=0.816, *p*=0.03.

b. Kaplan-Meier analyses of overall survival (OS, left) and event-free survival (EFS, right) in MM patients with high and low naïve $CD4^+$ T cells. Hazard ratios OS=1.22, *p*=0.10; Hazard ratios EFS=1.14, *p*=0.24.

c. Kaplan-Meier analyses of overall survival (OS, left) and event-free survival (EFS, right) in MM patients with high and low γδ T cells. Hazard ratios OS=0.869, *p*=0.45; Hazard ratios EFS=0.861, *p*=0.40.

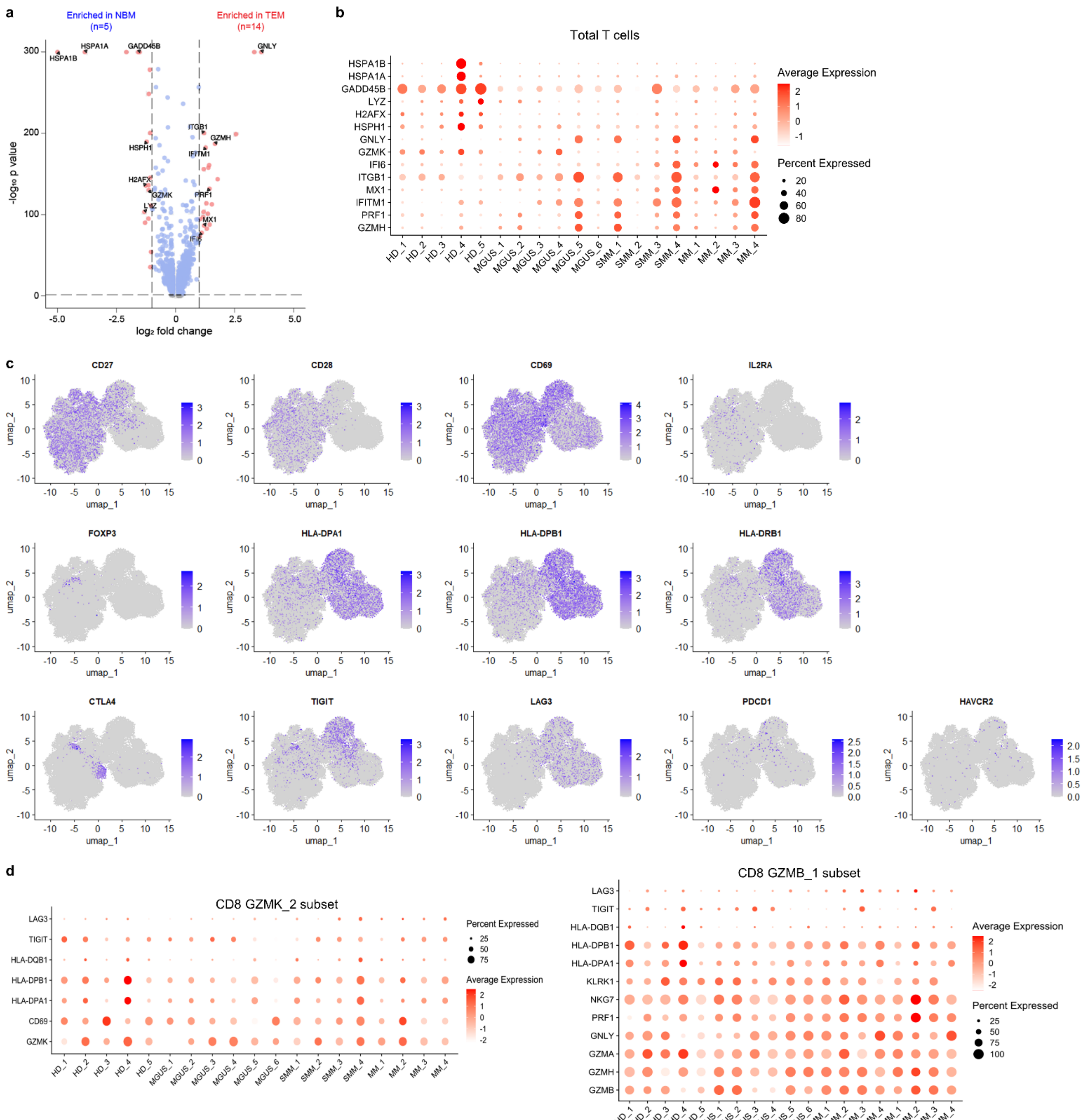

Supplemental Figure 8. CD8⁺ T cells do not show gene expression difference in MHC class II, cytotoxic and TIGIT within *GZMK*⁺ or *GZMB*⁺ subclusters among NBM and TME.

a. Volcano plot showing differential gene expression in T cells between normal bone marrow (NBM) and tumor microenvironment (TME).

b. Dot plot showing gene expression of T cells for individual patients and healthy donors.

c. Density map on the UMAP plot showing gene expression levels among T subtypes.

d. Dot plot showing gene expression of CD8 GZMK_2 cells (left) and CD8 GZMB_1 (right) for individual patients and healthy donors.

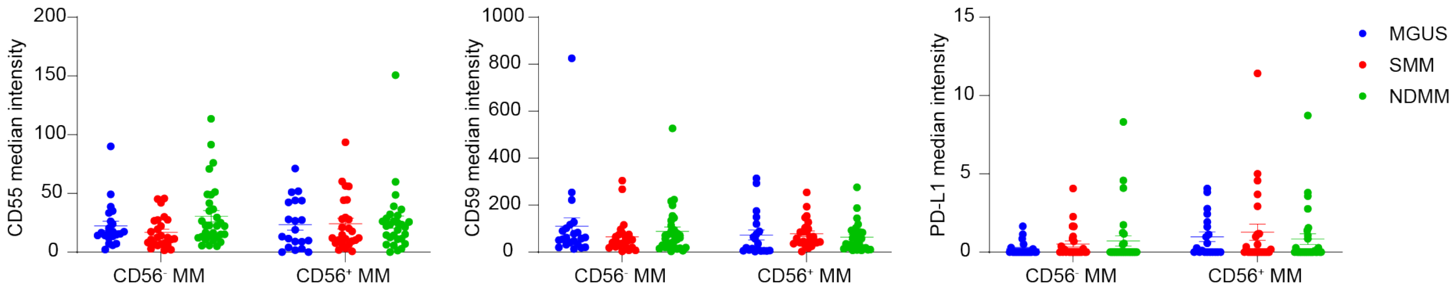

Supplemental Figure 9. The expression levels of CD56, CD59 and PD-L1 expression among MM clones had no change in MM compare with its precursor stages.

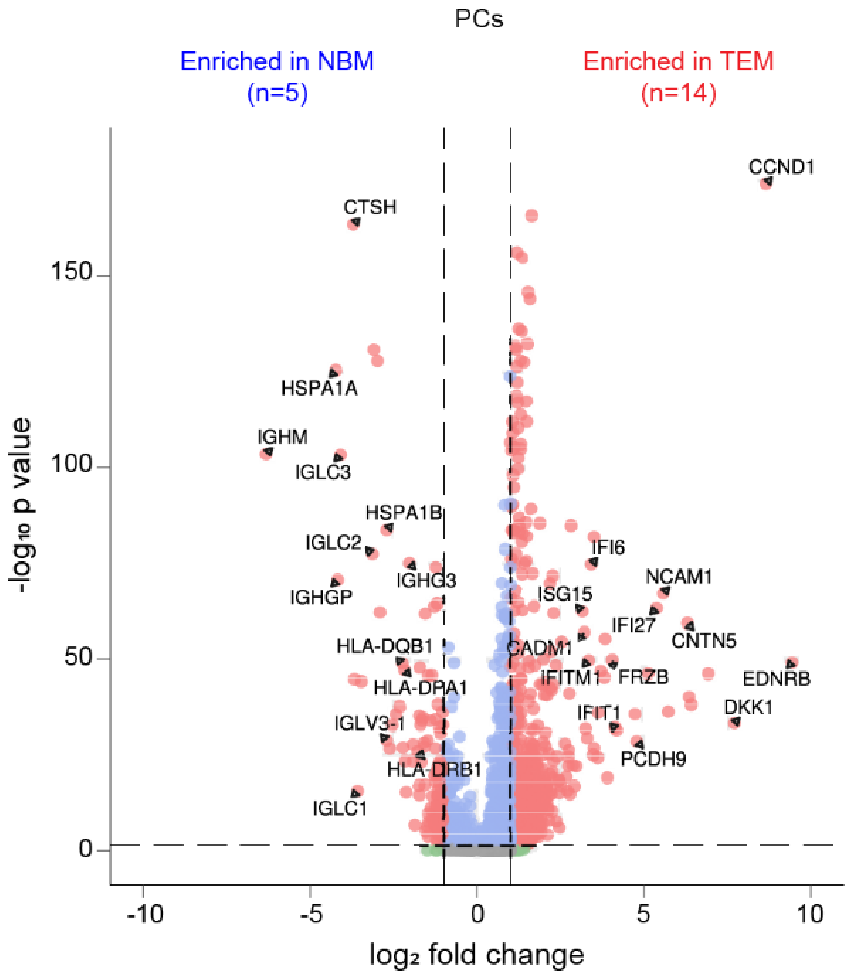

Supplemental Figure 10. Volcano plot showing differential gene expression in plasma cells between normal bone marrow (NBM) and tumor microenvironment (TME).

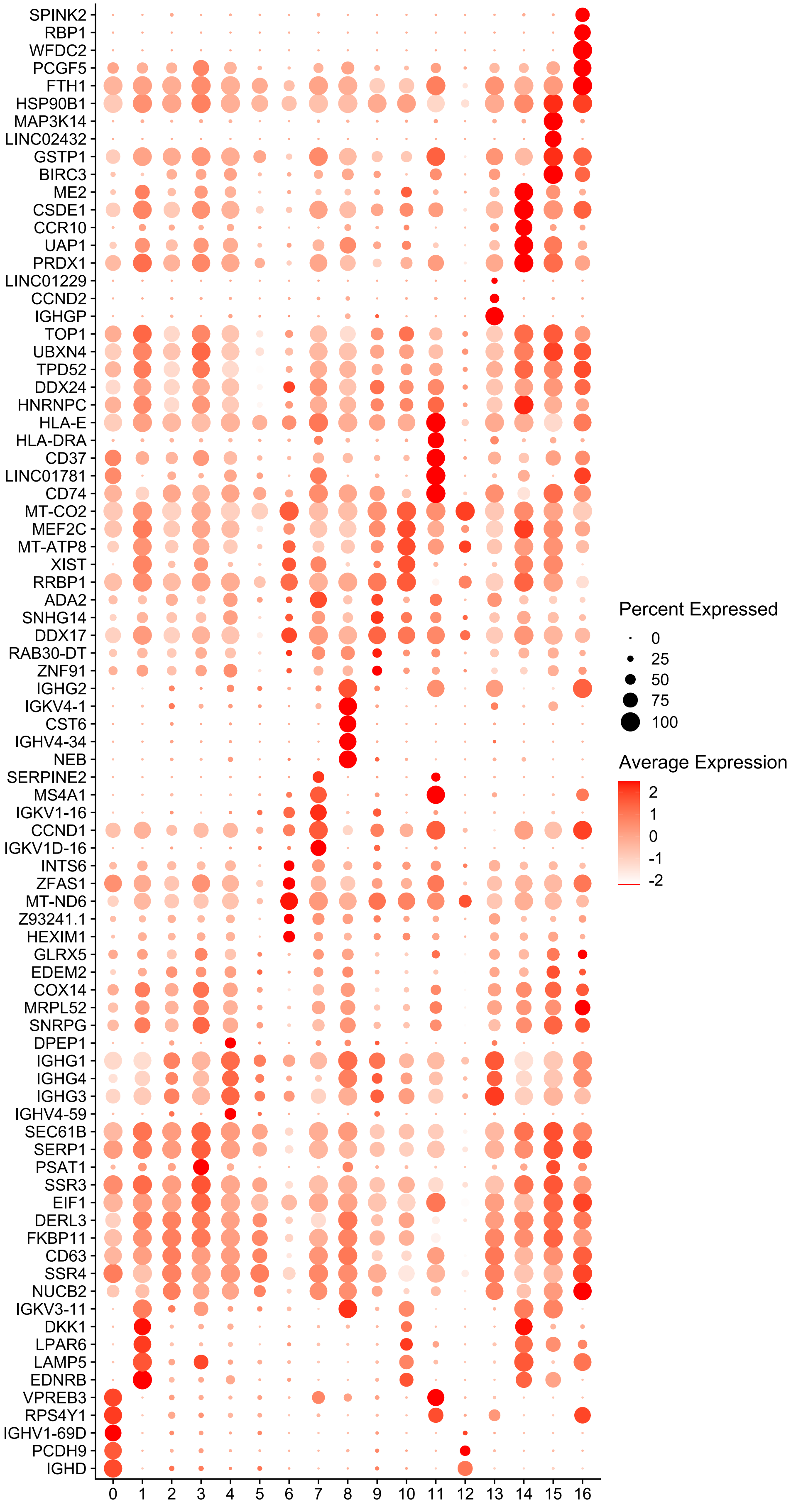

Supplemental Figure 11. Dot plot showing marker gene expression of plasma cell subtypes.

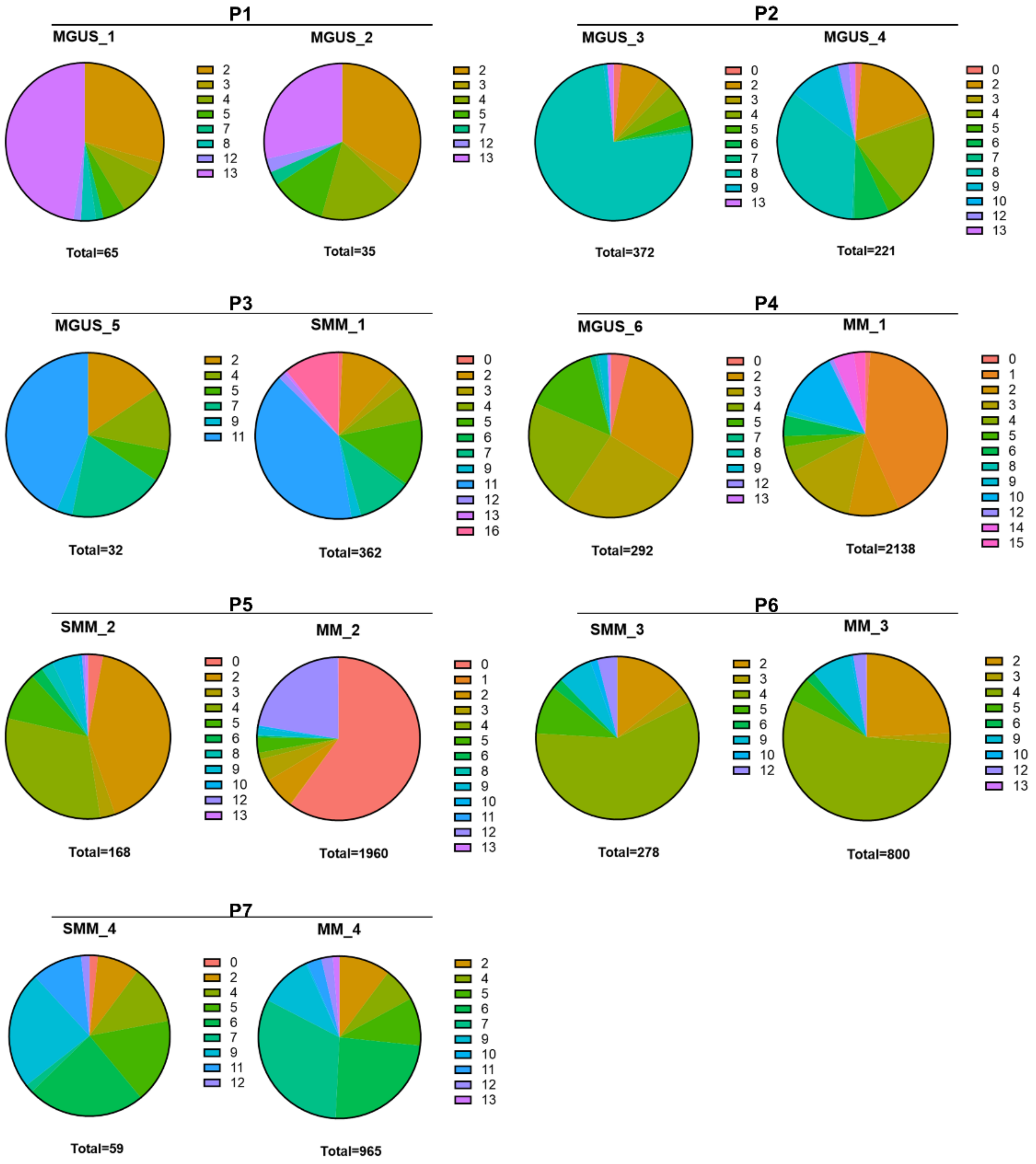

Supplemental Figure 12. Pie plots showing percentages of plasma cell subtypes in individual patients.

Supplemental Tables 1.

Clinical and laboratory characteristics of patients in the biopsy GEP project

Characteristics	MGUS (n=122)	SMM (n=122)	NDMM (n=704)
Age, median (range)	63 (33-88)	64 (34-82)	61 (25-84)
Male/female	63/59	67/55	415/289
ISS (I/II/III)	n/a	n/a	278/206/218
Immunoglobulin type			
IgA	9	19	141
IgD	0	1	15
IgM	0	1	1
IgG	47	92	388
Kappa light chain	39	72	428
Lambda light chain	19	49	254

Supplemental Table 2.

Clinical and laboratory characteristics of patients in the mass cytometry (CyTOF) project

Characteristics	MGUS (n=28)	SMM (n=30)	NDMM (n=33)
Age, median (range)	64 (43-95)	64 (34-81)	64 (38-82)
Male/female	11/17	17/13	18/15
ISS (I/II/III)	n/a	n/a	14/9/9
Immunoglobulin type			
IgA	3	6	7
IgD	0	1	0
IgM	1	0	0
IgG	19	19	16
Kappa light chain	15	18	10
Lambda light chain	9	12	13

Supplemental table 3.

Antibody panel used for mass cytometry staining

Target	Metal tag	Source
CD45	89Y	Fluidigm
CD3	154Sm	Fluidigm
CD4	176Yb	Fluidigm
CD8	141Pr	Fluidigm
CD45RA	143Nd	Fluidigm
CD25	149Sm	Fluidigm
FOXP3	162Dy	Fluidigm
CD27	155Gd	Fluidigm
CD28	160Gd	Fluidigm
CD127	168Er	Biolegend*
PD-1	174Yb	Fluidigm
CTLA-4	170Er	Fluidigm
TIGIT	153Eu	Fluidigm
TIM3	158Gd	Fluidigm
LAG3	150Nd	Fluidigm
CD56	166Er	Fluidigm
CD16	209Bi	Fluidigm
CD57	115In	Fluidigm
CD161	152Sm	Fluidigm
CD19	142Nd	Fluidigm
CD20	171Yb	Fluidigm
IgD	146Nd	Fluidigm
CD38	167Er	Fluidigm
CD14	175Lu	Fluidigm
CD15	164Dy	Fluidigm
CD11b	144Nd	Fluidigm
HLA-DR	173Yb	Fluidigm
CD33	163Dy	Biolegend*
CD11c	147Sm	Fluidigm
CD123	151Eu	Fluidigm
CD61	165Ho	Fluidigm
CD55	148Nd	Fluidigm
CD59	161Dy	Fluidigm
CCR6	145Nd	Fluidigm
CCR7	159Tb	Fluidigm
Ki67	172Yb	Fluidigm
CD24	169Tm	Fluidigm
PDL1	156Gd	Fluidigm

Note: * These antibodies were conjugated to their respective metal tags using the X8 polymer MaxPAR antibody conjugation kit (Fluidigm) according to the manufacturer’s protocol.

Supplemental Table 4.

Clinical and laboratory characteristics of patients participated in the scRNA-Seq project

Patient	Gender	Race	Immunoglobulin type	Light chain	Cytogenetics	MM subtypes
P1	Male	White / Caucasian	IgG	Kappa	t(14q32)	MF
P2	Female	White / Caucasian	IgG	Kappa	del(13q)	LB
P3	Male	White / Caucasian	IgG	Lambda	missing data	CD2
P4	Female	White / Caucasian	IgA	Kappa	missing data	HY
P5	Male	White / Caucasian	IgD	Kappa	del(17p13.1)	HY
P6	Male	White / Caucasian	IgG	Kappa	del(17p13.1)	HY
P7	Female	Black	IgG	Kappa	t(11;14)	CD2
